# Concordant Gene Expression in Leukemia Cells and Normal Leukocytes Is Associated with Germline *cis*-SNPs

**DOI:** 10.1371/journal.pone.0002144

**Published:** 2008-05-14

**Authors:** Deborah French, Wenjian Yang, Leo H. Hamilton, Geoffrey Neale, Yiping Fan, James R. Downing, Nancy J. Cox, Ching-Hon Pui, William E. Evans, Mary V. Relling

**Affiliations:** 1 Department of Pharmaceutical Sciences, St. Jude Children's Research Hospital, Memphis, Tennessee, United States of America; 2 Hartwell Center for Bioinformatics and Biotechnology, St. Jude Children's Research Hospital, Memphis, Tennessee, United States of America; 3 Department of Pathology, St. Jude Children's Research Hospital, Memphis, Tennessee, United States of America; 4 Department of Oncology, St. Jude Children's Research Hospital, Memphis, Tennessee, United States of America; 5 University of Tennessee, Memphis, Tennessee, United States of America; 6 Department of Human Genetics, University of Chicago, Chicago, Illinois, United States of America; National Cancer Institute at Frederick, United States of America

## Abstract

The degree to which gene expression covaries between different primary tissues within an individual is not well defined. We hypothesized that expression that is concordant across tissues is more likely influenced by genetic variability than gene expression which is discordant between tissues. We quantified expression of 11,873 genes in paired samples of primary leukemia cells and normal leukocytes from 92 patients with acute lymphoblastic leukemia (ALL). Genetic variation at >500,000 single nucleotide polymorphisms (SNPs) was also assessed. The expression of only 176/11,783 (1.5%) genes was correlated (p<0.008, FDR = 25%) in the two tissue types, but expression of a high proportion (20 of these 176 genes) was significantly related to *cis*-SNP genotypes (adjusted p<0.05). In an independent set of 134 patients with ALL, 14 of these 20 genes were validated as having expression related to *cis*-SNPs, as were 9 of 20 genes in a second validation set of HapMap cell lines. Genes whose expression was concordant among tissue types were more likely to be associated with germline *cis*-SNPs than genes with discordant expression in these tissues; genes affected were involved in housekeeping functions (*GSTM2*, *GAPDH* and *NCOR1*) and purine metabolism.

## Introduction

The influence of germline genetic variation on global gene expression in health[1]–[5]and disease[6]–[8] is an area of considerable interest. A number of recent studies have focused on the influence of germline variability in “normal” lymphoblastoid cell lines through association and linkage studies[9]–[13]. A considerable proportion of inter-individual differences in gene expression is likely due to germline variability produced by single nucleotide polymorphisms (SNPs). These SNPs may act by *cis* or by *trans* mechanisms[9], [11], [13], [14], although *cis*-regulatory variation appears to be more readily detectable than *trans*, with lower false positive findings[11], [14], [15]. Prior studies have focused on normal hematopoietic tissues and the majority are limited to the HapMap EBV-transformed lymphoblastoid cell lines instead of primary tissues. However, there are limited data indicating that in the absence of disease, leukocyte gene expression displays less variability within an individual than it does between individuals; differences partly attributable to gender and ethnicity[15]–[17]. A component of this invariant expression is produced by a subset of the genome comprising “housekeeping genes” that are continuously and ubiquitously expressed across multiple tissues and are likely essential for normal cellular function[18]–[21]. How germline polymorphisms in these housekeeping genes affect gene expression in multiple tissue types remains largely unexplored.

**Figure 1:**
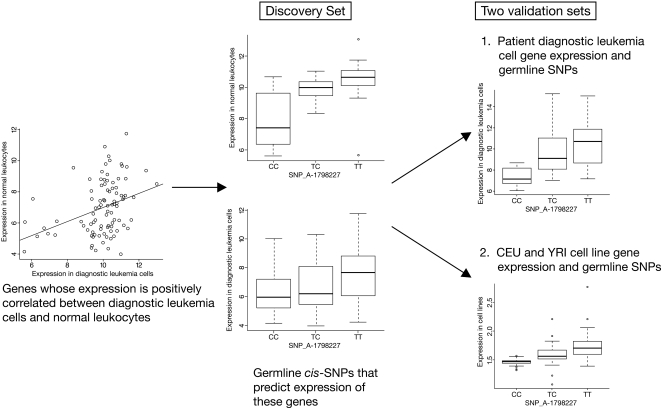
The overall strategy: Left: determination of genes whose expression is concordant between diagnostic leukemia cells and normal leukocytes; here the expression of *CTSW* is depicted as an example. Middle: discovery of germline *cis*-SNPs that are significantly related to the level of expression of such concordant genes; here a *cis*-SNP genotype is related to the expression of an example gene (*CTSW*). Right: validation of the relationship between these same *cis*-SNPs and their genes in two independent validation sets; here the same *cis*-SNP related to the expression of *CTSW*.

Tumor tissue gene expression is influenced not only by germline but also by somatically-acquired genomic variability. Inter-individual differences in gene expression within any given tumor type have been successfully used to refine tumor subtype classification and to identify genes associated with drug resistance and treatment outcome[22]–[25]. Although it is clear that some of the inter-individual variation in tumor tissue is due to somatically acquired changes specific to the cancer genome, it is not known to what extent this variation is influenced by differences in allele frequencies in germline polymorphisms among individuals. Herein we quantified gene expression from 92 patients with acute lymphoblastic leukemia (ALL) in paired normal and cancer tissues to assess the concordance of gene expression in these tissues, and the relationship between germline genomic variation and gene expression. Genotype-phenotype relationships were confirmed in an independent group of 134 patients with newly diagnosed ALL, and in the CEU and YRI populations of the HapMap cell lines (Figure 1).

## Results

### Gene expression that covaries in leukemia and normal cell primary tissues

We assessed gene expression for 11,783 genes in paired diagnostic leukemia cells and normal leukocytes (collected 8 weeks later) from 92 patients with newly diagnosed acute lymphoblastic leukemia (ALL). Unsupervised hierarchical clustering of gene expression produced two major groupings: diagnostic leukemia cells and normal leukocytes formed two discrete clusters (Figure S1), with 58% of the genes exhibiting discordance in gene expression (p<0.001) between the two primary tissue types. Within the diagnostic leukemia cell samples, clustering by leukemia subtype was observed, as we have previously described[22], [23]. The normal leukocyte samples did not segregate into any well-defined clusters based upon demographic factors (i.e. sex, race, and age).

The expression of 176 genes was significantly positively correlated between diagnostic leukemia cells and normal leukocyte samples (p<0.008), with a false discovery rate (FDR) of 25% (Table S1; Figures S2 and S3). There was no significant difference in the average expression levels of these 176 genes between the two tissues (p = 0.79, paired t-test).

### Genome-wide assessment of genotypes associated with concordant gene expression in leukemia cells and normal leukocytes (the discovery set)

**Figure 2:**
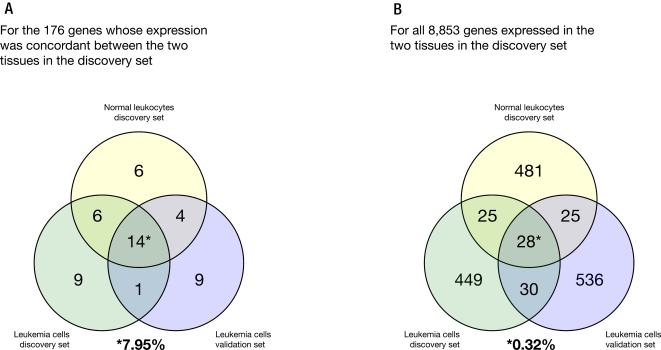
a) At adjusted p<0.05, there was no difference in the number of *cis*-SNPs associated with the expression of the concordant genes in the normal leukocytes and the leukemia cells in the discovery set. In the discovery set of normal leukocytes (yellow circle), there are 30 out of 176 genes that have significant *cis*-SNPs and in the discovery set of leukemia cells (green circle) there are 30 out of 176 genes that have significant *cis*-SNPs. Additionally, in the validation set of leukemia cells (blue circle) there are 28 out of 176 genes that have significant *cis*-SNPs. There are 14 out of 176 genes with significant c*is*-SNPs that are common to all 3 of the populations representing 7.95%^*^ of the 176 genes. b) At adjusted p<0.05, there was no difference in the number of *cis*-SNPs associated with the expression of all 8853 expressed genes in the normal leukocytes and the leukemia cells in the discovery set. In the discovery set of normal leukocytes (yellow circle), there are 559 genes with significant *cis*-SNPs and in the discovery set of leukemia cells (green circle), there are 532 genes that have significant *cis*-SNPs (p = 0.41, Fishers exact test). Additionally, in the validation set of leukemia cells (blue circle), there are 619 genes that have significant *cis*-SNPs. There are 28 out of 8853 genes with significant *cis*-SNPs that are common to all 3 populations representing 0.32%^*^ of the 8853 genes.

**Figure 3:**
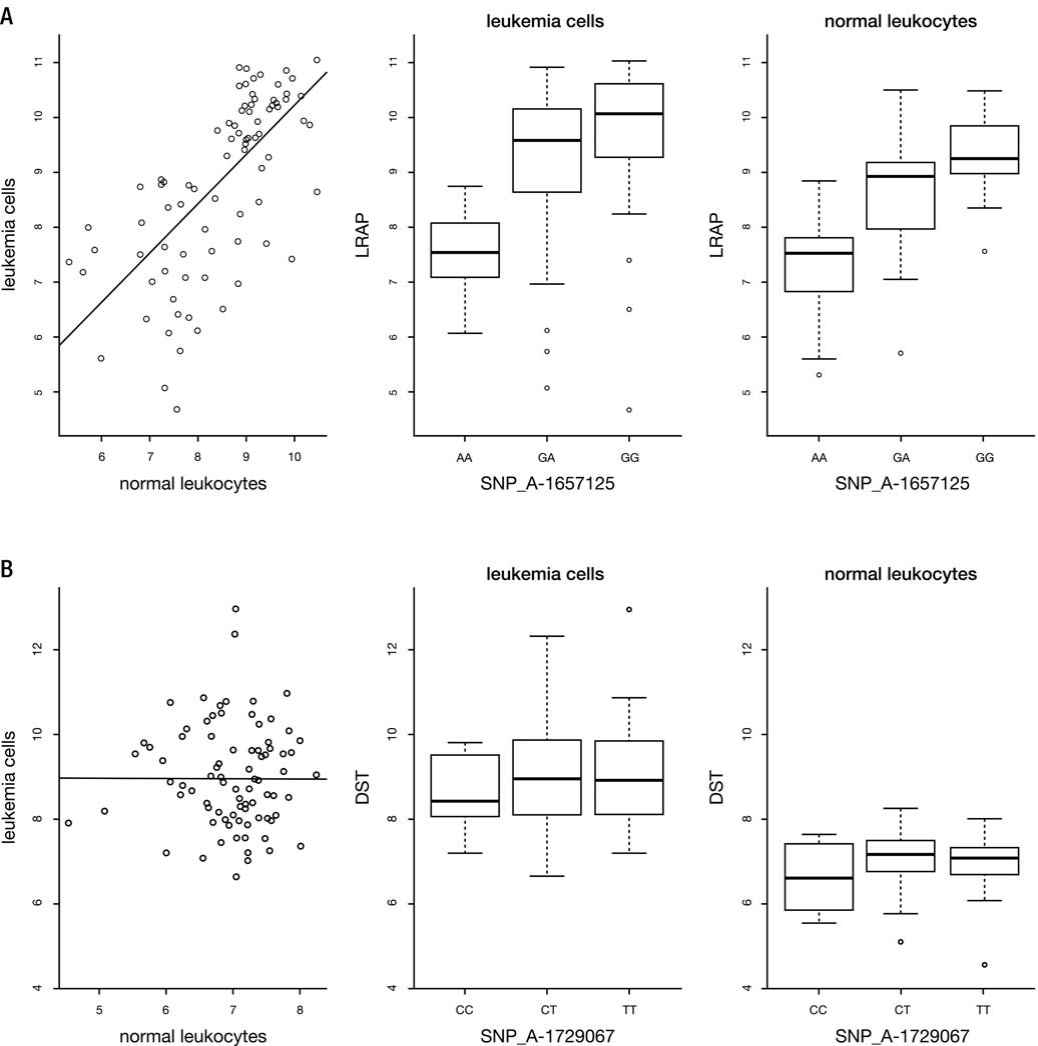
a) An example of one of the 176 genes (here *LRAP*) whose expression was concordant between diagnostic leukemia cells and normal leukocytes: Left: expression levels among 92 patients in leukemia cells and normal leukocytes are correlated (p = 1.11×10^−14^); Middle: expression of this gene (median, quartiles, range) in leukemia cells is associated (adjusted p = 0.0016) with the germline genotype of a particular *cis*-SNP in this gene; Right: expression of this gene in normal leukocytes is also associated (adjusted p = 0.0016) with the germline genotype at the same *cis*-SNP. (See Figure S4 for additional examples.) b) An example of one of the 8853 genes (here *DST*) whose expression was not concordant between diagnostic leukemia cells and normal leukocytes: Left: expression levels among 92 patients in leukemia cells and normal leukocytes are not correlated (p = 0.9384); Middle: expression of this gene in leukemia cells is not associated (adjusted p = 1.0000) with the germline genotype of a particular *cis*-SNP in this gene; Right: expression of this gene in normal leukocytes is also not associated (adjusted p = 0.9994) with the germline genotype at the same *cis*-SNP. (See Figure S4 for additional examples.)

**Figure 4:**
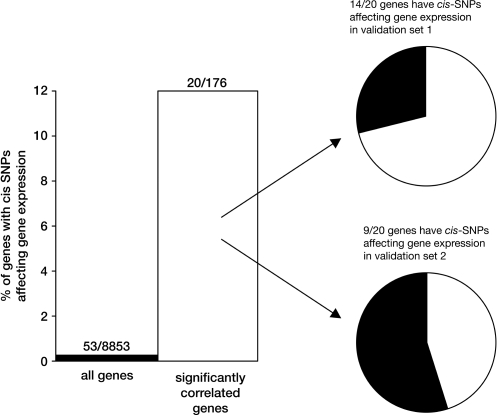
The 176 genes (open bars) whose expression was concordant between diagnostic leukemia cells and normal leukocytes were more likely (p = 3.71×10^−18^, Fishers exact test) to have *cis*-SNP genotypes (within 50 Kb of a gene) associated with their gene expression (20/176) than genes (closed bars) whose expression was discordant between the leukemia cells and normal leukocytes (53/8,853). A high proportion of these 176 genes whose expression was concordant between diagnostic leukemia cells and normal leukocytes in the discovery set also had *cis*-SNPs affecting gene expression in validation set 1 consisting of leukemia cells (14/20) and in validation set 2 consisting of HapMap lymphoid cell lines (9/20).

Of the 92 paired samples in the discovery set, 82 had germline genotypes available from a genome-wide scan of >500,000 SNPs. SNPs were evaluated for their influence on gene expression in both diagnostic leukemia cells and normal leukocytes independently, and these *cis*-SNPs that predicted gene expression in both leukemia cells and normal leukocytes were further examined. Genes whose expression was significantly positively correlated (p<0.008) between diagnostic leukemia cells and normal leukocytes were more likely (p = 3.71×10^−18^, Fishers exact test) to have *cis*-SNPs (within 50 Kb of a gene interrogated on the gene expression array used) whose allele frequency was significantly associated with gene expression than genes whose expression was discordant in leukemia cells and normal leukocytes (Figure 2). Specifically, only 53 of 8,853 (0.60%) commonly expressed genes (affected by 150 *cis*-SNPs) had *cis*-SNP genotypes that significantly predicted (adjusted p<0.05) gene expression in both diagnostic leukemia cell and normal leukocyte samples, compared to 20 of 176 (11.4%) genes (affected by 86 *cis*-SNPs) whose expression positively correlated (p<0.008) between diagnostic leukemia cells and normal leukocytes (Table 1; Figures 2, 3 and 4; Figure S4). We observed no difference in the number of genes with *cis*-SNPs associated with gene expression of the concordantly expressed genes between the leukemia cells (n = 30 genes) and the normal leukocytes (n = 30 genes) (Figure 2a). Of these 20 genes, the *cis*-SNPs associated with their expression found within the same gene were in high linkage disequilibrium (LD), but the SNPs associated with different genes were not in LD. There was no significant difference in the level of expression of these 20 genes between the two tissues (p = 0.49, paired t-test). The average expression level of these 20 genes was higher than the overall expression level of the 176 genes: only one out of the 20 genes (5%) had a gene expression level less than 8 in both tissues, compared to 58 genes out of the remaining 156 genes that correlated between tissue types (37%, p = 0.005) and 6,867 genes out of the other 11,697 (59%) transcripts that did not correlate between tissues (Figure S2). Additionally, a relatively high percentage of the observed variation in the expression of these 20 genes was attributed to the *cis*-SNPs, approximately 26% (range = 8–58%) in the leukemia cells and, 34% (range = 14–64%) in the normal leukocytes.

**Table 1:** 20 genes (21 probe sets) whose expression correlated between both diagnostic leukemia cell and normal leukocytes and was associated with *cis*-SNP genotypes (adjusted p<0.05).

Ensembl transcript ID	Gene Symbol	# of *cis*-SNPs interrogated per gene	Description	Adjusted p-value SNP vs expression in diagnostic leukemia cells	Adjusted p-value SNP vs expression in normal leukocytes	Diagnostic leukemia cell vs normal leukocyte expression correlation p-value
ENST00000343361^1^	LRAP^2^	24	leukocyte-derived arginine aminopeptidase	0.00160	0.00160	<0.00001
ENST00000336458	EIF5A	4	eukaryotic translation initiation factor 5A	0.02040	0.00760	<0.00001
ENST00000290649^1^	AMFR^3^	7	autocrine motility factor receptor	0.00100	0.00140	<0.00001
ENST00000304611^1^	PEX6^2^^,^ 3	4	peroxisomal biogenesis factor 6	0.00060	0.00060	<0.00001
ENST00000373238^1^	SAR1A^2^	10	SAR1 gene homolog A (S. cerevisiae)	0.00840	0.02080	<0.00001
ENST00000290921^1^	CTBP1	10	C-terminal binding protein 1	0.00100	0.00320	<0.00001
ENST00000216214^1^	FAM118A^2^^,^ 3	8	family with sequence similarity 118, member A	0.00080	0.00060	0.00001
ENST00000381633^1^	DDX17^3^	3	DEAD (Asp-Glu-Ala-Asp) box polypeptide 17	0.00700	0.00040	0.00001
ENST00000216019^1^	DDX17^3^	3	DEAD (Asp-Glu-Ala-Asp) box polypeptide 17	0.00280	0.00040	0.00005
ENST00000322244^1^	UBE1L2	16	ubiquitin-activating enzyme E1-like 2	0.00080	0.00360	0.00006
ENST00000307886^1^	CTSW^2^^,^ 3	1	cathepsin W	0.00620	0.00000	0.00040
ENST00000379221^1^	DNAJC15^3^	27	DnaJ (Hsp40) homolog, subfamily C, member 15	0.01400	0.03180	0.00136
ENST00000327435^1^	ADI1	9	acireductone dioxygenase 1	0.00140	0.00720	0.00254
ENST00000323013^1^	CTA-126B4.3^2^^,^ 3	2	CGI-96 protein	0.01460	0.00200	0.00274
ENST00000361151^1^	ZNF266^2^^,^ 3	11	zinc finger protein 266	0.00060	0.00040	0.00378
ENST00000355338^1^	WARS	15	tryptophanyl-tRNA synthetase	0.00500	0.01720	0.00415
ENST00000311014	DNAI2	8	dynein, axonemal, intermediate chain 2	0.01200	0.03860	0.00548
ENST00000328649	CIB1	4	calcium and integrin binding 1 (calmyrin)	0.00060	0.00020	0.00569
ENST00000340181	ETV7	2	ets variant gene 7 (TEL2 oncogene)	0.04360	0.04340	0.00589
ENST00000296581	LSM6	18	LSM6 homolog, U6 small nuclear RNA associated (S. cerevisiae)	0.00640	0.00720	0.00600
ENST00000291568	CSTB^2^^,^ 3	5	cystatin B (stefin B)	0.00140	0.00200	0.00716

1 14 of these 20 genes (15 of 21 probe sets) retained a significant *cis*-SNP regulation (adjusted p<0.05) in Validation Set 1.

Validation Set 2 used Affymetrix Exon Array® data^2^ and Illumina BeadChip array data^3^. Of the 20 genes present on the Exon Array and BeadChip array and in the discovery set, 8 and 9 retained significant *cis*-SNP associations respectively (adjusted p<0.05).

### Validation of association between gene expression and genotype

Validation set 1 consisted of an independent association analysis between diagnostic leukemia cell gene expression and germline SNPs from a separate group of 134 patients with newly diagnosed ALL. Amongst the 20 genes whose expression correlated in diagnostic leukemia cell and normal leukocyte paired samples and whose expression was associated with *cis*-SNP genotypes in the discovery set, 14 retained a significant *cis*-SNP association in validation set 1 (adjusted p<0.05) (Table 1; Figure 2a and Figure 4).

Validation in set 2 consisted of an independent association analysis between publicly available SNP and gene expression data from the 60 CEU and 60 YRI individuals that constitute the core unrelated members of the HapMap lymphoblastoid cell lines. Of the 20 concordantly expressed genes in the discovery set whose expression was associated with *cis*-SNP genotypes, all 20 were interrogated on the Affymetrix Exon Array (http://www.ncbi.nlm.nih.gov/geo/query/acc.cgiaccGSE7761), and the expression of 8 were also significantly associated with *cis*-SNP genotypes (Figure S5a); all 20 genes were also interrogated on the Illumina BeadChip array (http://www.ncbi.nlm.nih.gov/geo/query/acc.cgiaccGSE6536), and the expression of 9 were significantly associated with *cis*-SNP genotypes in validation set 2 (adjusted p<0.05) (Table 1; Figure 4). Thus, in two independent data sets, a high proportion of *cis*-SNPs associated with concordant gene expression in the discovery set were validated as also associating with gene expression in two independent data sets: the HapMap cell lines and another group of primary ALL blasts.

### Genes of interest

The 204 probe sets (Table S1) with expression positively correlating between diagnostic leukemia cells and normal leukocytes represent 176 distinct genes with diverse cellular functions. Several genes have important functions in system-wide homeostasis (e.g. *GSTM2* and *GAPDH*), while others are involved in protein synthesis (e.g. *RPS12*, *RPS4Y1 and RPL36AL*) or in purine metabolism (e.g. *GART*, *XDH*, *CANT1* and *ADSS*). Seven of these 176 genes overlap with those identified in the analysis of Whitney AR *et al.* that reported genes that show large inter-individual variation in expression in whole blood, including genes involved in RNA regulation (*DDX17*), nuclear protein import (*KPNA6*), and lymphocyte biology (*HLA-DQA1*, *CTSW*, and *GNLY*)[26]. The expression of *DDX17* was also shown to have strong evidence of a *cis*-acting transcriptional regulator in the analysis of Morley *et al.* using the CEU cell lines[9].

We found that a high percentage of genes (31/176 genes (18%)) whose expression correlated between paired tissue samples were among 1789 “housekeeping genes” (including *GSTM2*, *GAPDH* and *NCOR1*) that were previously found to be expressed across multiple tissues[20]. This is a much higher percentage compared to the overall reported percentage of “housekeeping genes” among all sampled genes (1789/22,685 or 0.7%) (p = 0.0001) (Table S2). Additionally, 5 of these “housekeeping genes” (*CIB1*, *CSTB*, *CTBP1*, *DDX17* and *WARS*) had *cis*-SNPs associated with their expression.

**Figure 5:**
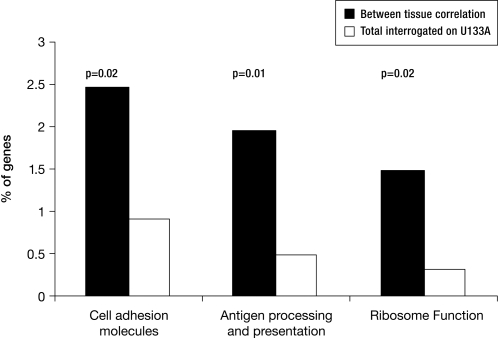
Genes whose expression is significantly associated between two tissue types (diagnostic leukemia cell and normal leukocytes) are overrepresented in specific pathways relative to all genes interrogated on the U133A microarray chip.

Among the 176 genes whose expression was concordant between the diagnostic leukemia cells and normal leukocytes, 157 were annotated to pathways in the KEGG database using the Database for Annotation, Visualization and Integrated Discovery (DAVID; http://david.abcc.ncifcrf.gov). These 157 genes are overrepresented in pathways involving antigen processing and presentation, cellular adhesion and ribosome function relative to all of the genes investigated on the Affymetrix HG-U133A Array® (Figure 5). Additional analysis of biological pathways of these 176 genes is provided in the Supporting Material (Figures S6 and S7).

### Sequence motifs for *cis*-SNPs

We queried the 100 bp flanking regions of the 86 *cis*-SNPs associated with the expression of the 20 genes whose expression was concordant between tissues using MEME and found 3 common motifs (Table S3). We then searched for these motifs among the 939 *cis*-SNPs associated with the expression of 532 genes in diagnostic leukemia cells (see Figure 2b, green circle), and the 1072 *cis*-SNPs associated with the expression of 559 genes in normal leukocyte samples (see Figure 2b, yellow circle). We found that the three motifs were significantly overrepresented in the 86 *cis*-SNP sequences compared to the other sequences (Table S3), however, these motifs are not known transcription factor binding sites, consistent with the fact that we found no pairs of genes with a significant correlation in expression in both the leukemia cells and the normal leukocytes (p<0.05, Pearson's correlation) (Figure S8 and Figure S9 respectively).

## Discussion

Although global gene expression has been related to germline genetic variation, this relationship has been based on associations between genotype and gene expression in a single tissue type sampled across multiple individuals. Herein we have identified a set of genes with concordant expression in two independent tissue types, leukemia cells and normal leukocytes, from the same individuals. We hypothesized that genes whose expression correlated in two independent tissues would be more likely to exhibit gene expression variation related to germline SNP genotypes than genes whose expression diverged between tissues. We indeed observed that genes with concordant gene expression in two tissue types were more likely to exhibit gene expression variation that was influenced by *cis*-germline genotype variation in primary tissue (discovery set), and these same *cis*-SNPs were associated with gene expression variation in two independent data sets, one comprising leukemia cells and one comprising non-malignant lymphoblastoid cell lines (Figure 4).

The significant biological differences between leukemia cells and normal peripheral blood leukocytes represent a rigorous setting in which to uncover such *cis* genetic regulation. Commonalities in gene expression and the underlying regulation of this expression in two different tissues (one with additional somatically acquired genetic defects, namely the leukemia cells) would be expected to be difficult to detect. The fact that we detected genes whose expression was concordant between tissue types that had strong evidence of *cis*-regulation, and that such *cis*-regulation was validated in two independent data sets, lends credence to our findings. To our knowledge, this is the largest group of individuals studied for genome-wide SNPs and genome-wide gene expression in two independent tissues.

Germline polymorphisms have long been associated with toxicity and outcome in cancer patients[27]–[29], but little has been reported about the possible effect of these polymorphisms on phenotypic variation in gene expression. Using a candidate gene approach, we have shown previously that germline copy number polymorphisms (in *GSTM1*, *TYMS*, and *UGT1A1*) were significantly associated with leukemia cell gene expression in patients with acute lymphoblastic leukemia, with the former two polymorphisms also being associated with risk of ALL relapse[29], [30]. The results from the genome-wide approach herein corroborate that germline SNP polymorphisms can also affect global gene expression profiles, in both leukemia cells and normal leukocytes. Our findings were validated in two independent validation sets, the first using 134 pediatric ALL diagnostic leukemia cell samples, the second using publicly available CEU and YRI lymphoblastoid cell line data.

We acknowledge that in both the patient and cell line validation sets, there are many more genes whose expression is significantly associated with *cis*-SNPs. However, our goal here was to determine if the *cis*-SNPs found to be associated with the concordant expression of genes in two independent tissues from the same individual could be replicated in other data sets, and we found this to be true.

The genes we found that correlated within an individual between diverse tissues had noteworthy overlap with genes whose expression predicted variation between individuals previously[2], [3], [26]. Prior studies have focused on elucidating genes whose expression differs both between individuals and within the same individual, using the same tissue type. In normal peripheral blood leukocytes[2], [3] and in whole blood[26], inter-individual gene expression variation was larger than intra-individual variation at multiple time points. Many of these genes are “housekeeping genes,” which could plausibly be expected to be expressed similarly across multiple tissues[20]. Genes that are members of the major histocompatibility complex (HLA) were also differentially expressed among individuals[2], [3], although the specific genes we identified differed from those previously reported. In our analysis, 86 *cis*-SNP genotypes were significantly associated with the expression of the 20 genes whose expression was concordant between two independent tissue types. Of these 86 SNPs, a high proportion (26/86 or 30%) was also significantly associated with inter-individual variation in gene expression in a prior interrogation of the HapMap cell lines[14].

Of the 20 genes with *cis*-SNP genotypes significantly associated with multiple tissue gene expression and that were confirmed in our first and second validation sets, one gene (*DDX17*) overlaps with those reported by Morley *et al.* as having strong *cis*-acting regulators (p<10^−9^)[9]. From a genome-wide scan, these authors noted 8 genes whose inter-individual variation in expression was strongly related to *cis* genetic variation in CEU lymphoblastoid cell lines; remarkably, 2 out of the 8 genes with strong *cis*-acting regulation were contained within our 20 genes.

The genes we observed whose expression is concordant between two discrete tissues within the same individual are involved in several cellular pathways and processes. Genes in overrepresented pathways include those involved in ubiquitously important cellular functions, including protein synthesis (*RPS12*, *RPS4Y1* and *RPL36AL*) and purine metabolism (*GART*, *XDH*, *CANT1* and *ADSS*). *RPS12* and *RPS4Y1* are components of the 40S subunit of the ribosome, while *RPL36AL* is a component of the 60S subunit, functioning together in the cytoplasm to catalyze protein synthesis.

We found that the 86 *cis*-SNPs associated with the expression of the 20 genes whose expression was concordant between tissues tended to be flanked by three DNA motifs that were overrepresented compared to those genes whose expression was not concordant and yet had *cis*-SNPs affecting their expression. However, there was no correlation between the expression of these 20 genes with each other within leukemia cells or within normal leukocytes, consistent with the fact that these 3 motifs are not known to represent the expression of DNA regulatory regions. This suggests that there may be an as yet unknown regulator of these genes.

Germline genetic polymorphisms may exert their effects on clinically important phenotypes via multiple mechanisms, only a fraction of which are related to their effects on transcript levels[31]. Some of these effects will be tissue specific (e.g. cytochrome P450 expression in liver) and others will have broad tissue distribution (e.g. thiopurine methyltransferase in liver, kidney, brain, leukemia cells, and erythrocytes)[32], [33]. Herein, using commonalities in expression between tissue types and evidence of *cis* regulation, we have identified a subset of genes whose expression is highly related to germline variation in multiple tissues.

## Materials and Methods

### Study Population

The discovery set consisted of 92 patients with newly diagnosed childhood acute lymphoblastic leukemia (ALL) enrolled on St. Jude Children's Research Hospital treatment protocol Total XV, who had both diagnostic leukemia cells and normal peripheral blood leukocytes available for expression array analysis. The Institutional Review Board at St. Jude Children's Research Hospital approved the investigation, and signed informed consent was obtained from patients or parents before enrollment, as appropriate. Samples were obtained at protocol-defined points; initial samples were obtained from diagnostic leukemia cells prior to the start of induction chemotherapy. After completion of 7 weeks of remission induction chemotherapy, but prior to beginning the consolidation phase of chemotherapy, a second sample was obtained, composed of unsorted normal peripheral blood leukocytes[34]. 82 of these 92 patients also had germline DNA samples available for genotyping analysis.

The first validation set comprised an additional 134 patients with newly diagnosed childhood ALL enrolled on St. Jude treatment protocols Total XIIIB and XV, with both diagnostic leukemia cell gene expression and germline SNP data available. The second validation set comprised lymphoblastoid cell lines derived from 60 unrelated Utah residents with ancestry from northern and Western Europe (CEU) and from 60 unrelated individuals from Yoruba in Ibadan, Nigeria (YRI) (www.hapmap.org).

### Gene expression profiling

We extracted RNA from diagnostic leukemia cells and normal peripheral blood leukocytes, assessed its integrity, generated labeled cRNA and analyzed the samples on the Affymetrix HG-U133A Arrays® as described[22]. The data were analyzed with Bioconductor 2.1 using the Affymetrix MAS5.0 algorithm (www.biocoductor.org). SNPs were downloaded from dbSNP build 126 if they were classed as “by-cluster,” “by-frequency,” “by-2hit-2allele,” or “by-hapmap[35]. Probes that encompassed any of these SNPs were excluded from analysis, and remaining probe sets with fewer than 3 probes were filtered out. Probes sets that may cross-hybridize with more than one genomic location were also filtered out[36], leaving 15,119 probe sets (annotated to 11,783 genes) available for further analysis. Gene expression signals were normalized so that all expression arrays had the same global average intensity. Gene expression data for CEU and YRI cell lines using the Affymetrix GeneChip® Human Exon 1.0 ST Array comprising ∼1.4 million probe sets, and using the Illumina Sentrix Human-6 Expression BeadChip Version 1 comprising around 14,500 probe sets, were downloaded from the following sites: http://www.ncbi.nlm.nih.gov/geo/query/acc.cgiaccGSE7761; http://www.ncbi.nlm.nih.gov/geo/query/acc.cgiaccGSE6536 [14], [37].

### Genotyping

High quality germline DNA was extracted from the normal leukocytes. A total of 250 ng of DNA was digested separately with the *XbaI* and *HindIII* restriction enzymes and then amplified, labeled and hybridized to the Affymetrix GeneChip® Human Mapping 100 K and 500 K Array Sets. The chips were scanned, and genotype calls were made using the BRLMM algorithm as implemented in the GTYPE software (http://www.affymetrix.com/products/software/specific/gtype.affx). SNPs with call rates of less than 95% in all patients were filtered out, and only SNPs within 50 Kb of a gene represented on the U133A expression array were included, leaving 176,561 SNPs for further analysis. Genotypes for the SNPs represented on the Affymetrix GeneChip® Human Mapping 100 K and 500 K Array Sets of the CEU and YRI cell lines were downloaded from release 22 on the International HapMap project website (www.hapmap.org).

### Data analysis

Spearman's rho rank correlation was used to estimate the correlation in gene expression between diagnostic leukemia cells and normal leukocytes for each probe set. The false discovery rate (FDR) was estimated based on the q-value method[38].

Hierarchical clustering was applied to the normalized gene expression signals of the diagnostic leukemia cells and the normal leukocytes in order to cluster samples based on the probe sets whose expression was most highly correlated between these two tissue types.

The association between the gene expression signal of each probe set and each *cis*-SNP in the diagnostic leukemia cells, in the normal leukocytes, and in the cell lines (independently), was evaluated based on linear regression using a codominant model. That is, AA genotypes were coded as 0, AB genotypes were coded as 1 and BB genotypes were coded as 2 [11]. We adjusted for race by including race as a covariate in the regression model.

We first computed the observed p-values for the association for each SNP genotype and the gene expression for the number of SNPs (N) within that particular gene. In order to account for the multiple testing problem caused by multiple SNPs within a gene, we performed 5,000 permutations, by randomly permuting the gene expression values and recomputing the association p-values between each SNP and the gene expression level. For each permutation, we recorded the minimum permuted p-value among the N permuted p-values for a gene and compared the observed p-values to the minimum p-value obtained from the permutation to adjust for multiple testing. The adjusted p-value for each SNP is the percentage of permutations with the observed p-value ≥ the minimum permuted p-value and takes into account the different number of SNPs per gene. This procedure was utilized for all analyses reported herein (adjusted p<0.05) [39]. There was no difference in the number of *cis*-SNPs per gene in the 20 genes whose expression was concordant between tissues and that had *cis*-SNPs associated with their expression, compared to the number of *cis*-SNPs per gene for all of the genes (n = 8853) that were expressed in both the leukemia cells and the normal leukocytes (Figure S10).

In our validation analysis, we determined the *cis*-SNPs that were associated with the expression of a gene independently in the diagnostic leukemia cells (validation set 1) and cell lines (validation set 2). We then looked for overlap in the *cis*-SNP/genes in these validation sets with our discovery population by matching the *cis*-SNP/gene pairs with adjusted p<0.05.

### Bioinformatics

We queried the Database for Annotation, Visualization and Integrated Discovery (DAVID; http://david.abcc.ncifcrf.gov) to assess the function and pathways of the 176 genes whose expression was concordant between tissues, the 20 genes of these 176 that had *cis*-SNPs significantly associated with their expression and the 1574 genes whose expression was not concordant between these tissues, but that had *cis*-SNPs predicting their expression in the normal leukocytes and the diagnostic leukemia cell samples from the discovery set and from the validation set (Figure 2b) [40].

We retrieved the 100 bp flanking sequences for each of the *cis*-SNPs using BioMart (http://www.biomart.org/), and masked repetitive DNA elements using the RepeatMask program (http://repeatmasker.org/cgi-bin/WEBRepeatMasker). Using MEME (Multiple Em for Motif Elicitation; http://meme.sdsc.edu/), we searched for repeated sequence patterns, or motifs, that occurred in these DNA sequences [41]. To search for these motifs in other *cis*-SNP sequences, we used MAST (Motif Alignment and Search Tool), a companion tool of MEME. We used TRANSFAC® (the gene transcription factor database) to determine if any of the common flanking motifs were known *cis*- or *trans*-acting regulatory DNA elements.

## Supporting Information

Table S1 176 genes (204 probe sets) whose expression was concordant between diagnostic leukemia cells and normal leukocytes (p<0.008).
(0.37 MB DOC)

Table S2 31 “housekeeping genes” whose expression is concordant between diagnostic leukemia cells and normal peripheral blood leukocytes
(0.07 MB DOC)

Table S3 Using MEME, 3 DNA sequence motifs were found to be overrepresented in the 20 genes whose expression was concordant between leukemia cells and normal leukocytes and that had cis-SNPs associated with their expression. These motifs were also found in the genes in the leukemia cells (green shading) and normal leukocytes (yellow shading) that had cis-SNPs associated with their expression, but whose expression was not concordant in these two tissues. None of the motifs are known transcription factor binding sites.
(0.03 MB DOC)

Figure S1 Unsupervised hierarchical clustering of gene expression for 184 samples (columns) from 92 patients indicates two distinct clusters which segregate the different tissue types: diagnostic leukemia cells and normal leukocytes. (Blood: normal leukocyte samples; BH: B-lineage ALL with hyperdiploid karyotype; BN: B-lineage ALL with non-hyperdiploid karyotype; bcr: BCR-ABL translocation; e2a: E2A-PBX translocation; mll: MLL-AF4 fusion; other: B-lineage ALL with no defined translocations; tel: TEL-AML1 translocation; T: T-lineage ALL).
(0.05 MB DOC)

Figure S2 Each circle represents a probe set. The average expression of each probe set in diagnostic leukemia cells for the 92 patients is shown on the x-axis and for normal leukocytes is shown on the y-axis. The line of identity shows comparable expression levels in both cell types. The blue circles indicate the 204 probe sets whose expression was concordant between the diagnostic leukemia cells and the normal leukocytes. The red circles indicate the 21 of these 204 probe sets that had cis-SNPs predicting their expression. The grey circles are the remaining 14,915 probe sets. The closer the circles are to the line of identity, the more similar the level of expression of that probe set in both tissues. The dotted line indicates a log2 expression level of 8, indicating that the 21 probe sets with cis-SNPs predicting their expression is higher than the overall expression of the 204 probe sets whose expression is concordant between tissue types.
(2.06 MB DOC)

Figure S3 a) Histogram showing the number of probe sets (y-axis) whose expression was correlated between tissue types at specific p-value cut offs (x-axis). The histogram indicates a significant correlation between gene expression in diagnostic leukemia cells and normal leukocytes as shown by an over-representation of probe sets with low p-values. A positive correlation of gene expression between diagnostic leukemia cells and normal leukocytes is shown by white shading and a negative correlation is shown by grey shading. The figure indicates a significantly higher number of probe sets than expected are positively correlated between diagnostic leukemia cells and normal leukocytes. b) Hierarchical clustering of gene expression with each column representing one of the 184 patient samples (92 leukemia cell and 92 normal leukocyte samples) and each row representing one of the top 136 probe sets (ordered by p-value; p<0.0048 cut-off) that are positively correlated between the diagnostic leukemia cells and normal leukocytes. Using these 136 probe sets, 55 out of the 92 patient sample pairs were clustered together as indicated with blue dashes below the clustering. Green = low expression and red = high expression.
(0.12 MB DOC)

Figure S4 a) Examples of genes (here ADI1, PEX6, AMFR, ZNF266 and SAR1A) whose expression was concordant between diagnostic leukemia cells and normal leukocytes: Left: expression levels among 92 patients in leukemia cells and normal leukocytes are correlated (p = 0.0025, <0.00001, 1.44×10−8, 0.0038, <0.00001 respectively); Middle: expression of these genes (median, quantiles, range) in leukemia cells was associated (adjusted p = 0.0016, 0.0006, 0.0002, 0.0004, 0.001 respectively) with the germline genotype of a particular cis-SNP in this gene; Right: expression of these genes in normal leukocytes was associated (adjusted p = 0.0072, 0.0006, 0.0014, 0.0004, 0.0208 respectively) with the germline genotype at the same cis-SNP. b) Examples of genes (here NRG1, NCOA1, C20orf23, C9orf39, ATM) whose expression was not concordant between diagnostic leukemia cells and normal leukocytes: Left: expression levels among 92 patients in leukemia cells and normal leukocytes was not concordant (p = 0.9799, 0.9377, 0.9523, 0.9128, 0.9607 respectively); Middle: expression of these genes in leukemia cells was not associated (adjusted p = 0.9996, 0.080, 1.000, 0.5106, 0.9996 respectively) with the germline genotype of a particular cis-SNP in this gene; Right: expression of these genes in normal leukocytes was not associated (adjusted p = 1.000, 1.000, 1.000, 0.9982, 0.9320 respectively) with the germline genotype at the same cis-SNP.
(0.20 MB DOC)

Figure S5 a) At adjusted p<0.05, there was no difference in the number of cis-SNPs associated with the expression of the 176 concordant genes in the normal leukocytes and the leukemia cells that constituted our discovery set. 133 of these 176 concordantly expressed genes are represented on the Exon Array for the HapMap cell lines that constituted one validation set. There was no significant difference between the number of cis-SNPs associated with the expression of these 133 genes in the HapMap cell lines (validation set) compared to the number of cis-SNPs associated with expression of the 176 genes in the normal leukocytes (p = 0.34, Fishers exact test) or the diagnostic leukemia cells (p = 0.34, Fishers exact test). Specifically, in the normal leukocytes (yellow circle), there are 30 genes with significant cis-SNPs; in the leukemia cells (green circle), there are 30 genes with significant cis-SNPs; and in the HapMap cell lines (pink circle), there are 17 genes with significant cis-SNPs, focusing on the genes whose expression was concordant between leukemia cells and normal leukocytes. There are 8 out of 176 genes (centre of VENN diagram) with significant cis-SNPs that are common to all 3 sets representing 4.5%* of the 176 genes. b) At adjusted p<0.05, there was no difference in the number of cis-SNPs associated with the expression of the 8,853 expressed genes in the normal leukocytes and the leukemia cells that constituted one discovery set. 8,187 of the 8,853 expressed genes are represented on the Exon Array for the HapMap cell lines that constituted our validation set. There was a significant difference between the number of cis-SNPs associated with the expression of these 8,187 genes in the HapMap cell lines (validation set) compared to the number of cis-SNPs associated with expression of the 8,853 genes in the normal leukocytes (p = 1.30×10−11, Fishers exact test) and the diagnostic leukemia cells (p = 3.00×10−14, Fishers exact test). Specifically, in the normal leukocytes (yellow circle), there are 559 genes with significant cis-SNPs; in the leukemia cells (green circle), there are 532 genes with significant cis-SNPs; and in the HapMap cell lines (pink circle), there are 743 genes with significant cis-SNPs, whose expression was not concordant between tissues. There are 15 out of 8,853 genes (center of VENN diagram) with significant cis-SNPs common to all 3 sets representing 0.17%* of the 8,853 genes.
(1.46 MB DOC)

Figure S6 Results of pathway analysis of genes whose expression was concordant between leukemia cells and normal leukocytes and had cis-SNPs associated with their expression in the discovery set (n = 20) compared to those genes that are expressed, but not concordantly expressed in the two tissues and have cis-SNPs associated with their expression (n = 1574), as determined by interrogating the Database for Annotation, Visualization and Integrated Discovery (DAVID; http://david.abcc.ncifcrf.gov/). None of the pathways reached statistical significance using this tool.
(0.08 MB DOC)

Figure S7 Results of pathway analysis of genes whose expression was concordant between leukemia cells and normal leukocytes in the discovery set and had cis-SNPs affecting their expression (n = 20) to over represent specific pathways compared to those genes that did not have cis-SNPs associated with their expression (n = 156) as determined by interrogating DAVID. Genes involved in the tryptophan metabolism pathway were marginally significantly over represented using this tool (p = 0.05, Fishers exact test).
(0.07 MB DOC)

Figure S8 There was no strong correlation (p>0.05, Pearson's correlation) in the diagnostic leukemia cells of expression between the 20 cis-SNP genes whose expression is concordant between diagnostic leukemia cells and normal leukocytes. Yellow indicates a strong positive correlation and increasing blue indicates no strong positive correlation.
(0.10 MB DOC)

Figure S9 There was no strong correlation (p>0.05, Pearson's correlation) in the normal leukocytes of expression between the 20 cis-SNP genes whose expression is concordant between diagnostic leukemia cells and normal leukocytes. Yellow indicates a strong positive correlation and increasing blue indicates no strong positive correlation.
(0.10 MB DOC)

Figure S10 There was no significant difference between the number of cis-SNPs per gene in the 20 genes whose expression was concordant between tissue types and that had cis-SNPs associated with their expression and the 8,853 genes whose expression was not concordant (p = 0.769, Wilcoxon test).
(0.06 MB DOC)
