# Supplementary material for: Concordant Gene Expression in Leukemia Cells and Normal Leukocytes Is Associated with Germline cis-SNPs
Source: PLoS One. 2008 May 14;3(5):e2144. doi: 10.1371/journal.pone.0002144 (PMC2374895; doi:10.1371/journal.pone.0002144)
Supplement: Table S1 — 176 genes (204 probe sets) whose expression was concordant between diagnostic leukemia cells and normal leukocytes (p<0.008). (0.37 MB DOC) [file pone.0002144.s001.doc]

Table S1: 176 genes (204 probe sets) whose expression was concordant between diagnostic leukemia cells and normal leukocytes (p<0.008).

| **Ensembl Transcript ID** | **p-value** | **Rho** | **Gene Symbol** | **Chromosome** | **Band** |
| --- | --- | --- | --- | --- | --- |
| ENST00000250784 | 0.00000 | 0.70837 | *RPS4Y1* | Y | p11.31 |
| ENST00000317961 | 0.00000 | 0.69815 | *JARID1D* | Y | q11.222 |
| ENST00000343361 | 0.00000 | 0.69749 | *NP_071745.1* | 5 | q15 |
| ENST00000382772 | 0.00000 | 0.68420 | *EIF1AY* | Y | q11.223 |
| ENST00000336079 | 0.00000 | 0.68049 | *DDX3X* | X | q11.21 |
| ENST00000360160 | 0.00000 | 0.66352 | *DDX3X* | X | q11.21 |
| ENST00000361365 | 0.00000 | 0.65963 | *EIF1AY* | Y | q11.223 |
| ENST00000338981 | 0.00000 | 0.61662 | *USP9Y* | Y | q11.21 |
| ENST00000362096 | 0.00000 | 0.61663 | *UTY* | Y | q11.21 |
| ENST00000230050 | 0.00000 | 0.60161 | *RPS12* | 6 | q23.2 |
| ENST00000329134 | 0.00000 | 0.58367 | *UTY* | Y | q11.21 |
| ENST00000336458 | 0.00000 | 0.57713 | *EIF5AP1* | 17 | p13.1 |
| ENST00000336452 | 0.00000 | 0.57101 | *EIF5AP1* | 17 | p13.1 |
| ENST00000290649 | 0.00000 | 0.54922 | *AMFR* | 16 | q12.2 |
| ENST00000343139 | 0.00000 | 0.52148 | *HLA-DQA1* | 6 | p21.32 |
| ENST00000331397 | 0.00000 | 0.50961 | *UTY* | Y | q11.21 |
| ENST00000304611 | 0.00000 | 0.50386 | *PEX6* | 6 | p21.1 |
| ENST00000359712 | 0.00000 | 0.49623 | *ITGB1BP1* | 2 | p25.1 |
| ENST00000334668 | 0.00000 | 0.49051 | *HLA-F* | 6 | p22.1 |
| ENST00000373238 | 0.00000 | 0.47234 | *SAR1A* | 10 | q22.1 |
| ENST00000360270 | 0.00000 | 0.47108 | *MSN* | X | q12 |
| ENST00000383251 | 0.00000 | 0.46935 | *HA23* | 6 |  |
| ENST00000287701 | 0.00000 | 0.46898 | *HMBOX1* | 8 | p21.1 |
| ENST00000373625 | 0.00000 | 0.46843 | *KPNA6* | 1 | p35.1 |
| ENST00000290921 | 0.00000 | 0.45940 | *CTBP1* | 4 | p16.3 |
| ENST00000336112 | 0.00001 | 0.45228 | *ASXL2* | 2 | p23.3 |
| ENST00000285093 | 0.00001 | 0.44771 | *ACAA2* | 18 | q21.1 |
| ENST00000216214 | 0.00001 | 0.44326 | *FAM118A* | 22 | q13.31 |
| ENST00000381633 | 0.00001 | 0.44184 | *DDX17* | 22 | q13.1 |
| ENST00000333628 | 0.00003 | 0.42088 | *TUBB2A* | 6 | p25.2 |
| ENST00000331666 | 0.00003 | 0.42046 | *EIF3S8* | 16 | p11.2 |
| ENST00000302303 | 0.00004 | 0.41669 | *RFX3* | 9 | p24.2 |
| ENST00000058691 | 0.00004 | 0.41473 | *HEBP2* | 6 | q23.3 |
| ENST00000382089 | 0.00004 | 0.41305 | *ADI1* | 2 | p25.3 |
| ENST00000304003 | 0.00005 | 0.41139 | *Q29927* | 6 |  |
| ENST00000216019 | 0.00005 | 0.40957 | *DDX17* | 22 | q13.1 |
| ENST00000374943 | 0.00005 | 0.40874 | *HB25* | 6 | p21.32 |
| ENST00000296754 | 0.00006 | 0.40730 | *ARTS1* | 5 | q15 |
| ENST00000244546 | 0.00006 | 0.40681 | *PEX6* | 6 | p21.1 |
| ENST00000322244 | 0.00006 | 0.40461 | *UBE1L2* | 4 | q13.2 |
| ENST00000326729 | 0.00008 | 0.40023 | *GSTM2* | 1 | p13.3 |
| ENST00000376803 | 0.00010 | 0.39346 | *HLA-G* | 6 | p22.1 |
| ENST00000263265 | 0.00011 | 0.39235 | *PLEKHA4* | 19 | q13.33 |
| ENST00000362758 | 0.00011 | 0.39172 | *PRKY* | Y | p11.2 |
| ENST00000320955 | 0.00012 | 0.38956 | *SPTBN5* | 15 | q15.1 |
| ENST00000310838 | 0.00013 | 0.38927 | *Q9P007* | 1 | p36.12 |
| ENST00000253320 | 0.00016 | 0.38340 | *CYorf15B* | Y | q11.222 |
| ENST00000268712 | 0.00020 | 0.37853 | *NCOR1* | 17 | p11.2 |
| ENST00000263549 | 0.00025 | 0.37318 | *PARP12* | 7 | q34 |
| ENST00000367439 | 0.00027 | 0.37142 | *GLRX2* | 1 | q31.2 |
| ENST00000360896 | 0.00031 | 0.36819 |  | 20 | q11.23 |
| ENST00000238091 | 0.00031 | 0.36760 | *ITGB1BP1* | 2 | p25.1 |
| ENST00000359353 | 0.00032 | 0.36718 | *DDX42* | 17 | q23.3 |
| ENST00000383507 | 0.00038 | 0.36290 | *HLAG* | 6 |  |
| ENST00000273048 | 0.00039 | 0.36205 | *C2orf17* | 2 | q35 |
| ENST00000307886 | 0.00040 | 0.36127 | *CTSW* | 11 | q13.1 |
| ENST00000188403 | 0.00049 | 0.35653 | *NR1H4* | 12 | q23.1 |
| ENST00000324695 | 0.00050 | 0.35573 | *TRPM8* | 2 | q37.1 |
| ENST00000273814 | 0.00054 | 0.35391 | *DGKQ* | 4 | p16.3 |
| ENST00000307771 | 0.00058 | 0.35210 | *ZRSR1* | 5 | p22.2 |
| ENST00000376816 | 0.00069 | 0.34743 | *HLA-G* | 6 | p22.1 |
| ENST00000289823 | 0.00075 | 0.34514 | *FGF17* | 8 | p21.3 |
| ENST00000293362 | 0.00075 | 0.34514 | *PSME3* | 17 | q21.31 |
| ENST00000388749 | 0.00098 | 0.33818 | *HNRPL* | 19 | q13.2 |
| ENST00000361450 | 0.00100 | 0.33762 | *PPP2R3B* | X | p22.33 |
| ENST00000228820 | 0.00101 | 0.33721 | *PARP11* | 12 | p13.32 |
| ENST00000383100 | 0.00105 | 0.33616 | *HB2S* | 6 |  |
| ENST00000240333 | 0.00106 | 0.33600 | *SLC35B1* | 17 | q21.33 |
| ENST00000229239 | 0.00120 | 0.33252 | *GAPDH* | 12 | p13.31 |
| ENST00000329522 | 0.00126 | 0.33118 |  | 14 | q24.3 |
| ENST00000263863 | 0.00133 | 0.32962 | *GNLY* | 2 | p11.2 |
| ENST00000379221 | 0.00136 | 0.32902 | *DNAJC15* | 13 | q14.11 |
| ENST00000302345 | 0.00137 | 0.32882 | *CANT1* | 17 | q25.3 |
| ENST00000336216 | 0.00139 | 0.32847 | *HMG20A* | 15 | q24.3 |
| ENST00000313071 | 0.00142 | 0.32791 | *FOXG1B* | 14 | q12 |
| ENST00000286437 | 0.00151 | 0.32616 | *AFF2* | X | q28 |
| ENST00000306576 | 0.00158 | 0.32482 | *ZNF672* | 1 | q44 |
| ENST00000261479 | 0.00161 | 0.32441 | *PSMA6* | 14 | q13.2 |
| ENST00000339300 | 0.00162 | 0.32420 | *CANT1* | 17 | q25.3 |
| ENST00000309451 | 0.00167 | 0.32321 | *FAM128A* | 2 | q21.1 |
| ENST00000314566 | 0.00170 | 0.32286 | *AMFR* | 16 | q12.2 |
| ENST00000306562 | 0.00172 | 0.32252 | *ZNF672* | 1 | q44 |
| ENST00000267512 | 0.00176 | 0.32176 | *RAB15* | 14 | q23.3 |
| ENST00000369836 | 0.00181 | 0.32107 | *GSTM2* | 1 | p13.3 |
| ENST00000262607 | 0.00181 | 0.32099 | *CECR1* | 22 | q11.1 |
| ENST00000318158 | 0.00186 | 0.32019 | *GRHPR* | 9 | p13.2 |
| ENST00000349769 | 0.00193 | 0.31914 | *CPSF1* | 8 | q24.3 |
| ENST00000205061 | 0.00203 | 0.31763 | *GLG1* | 16 | q22.3 |
| ENST00000238571 | 0.00206 | 0.31722 | *YLPM1* | 14 | q24.3 |
| ENST00000054668 | 0.00207 | 0.31719 | *UTS2* | 1 | p36.23 |
| ENST00000347890 | 0.00208 | 0.31691 | *HPSE2* | 10 | q24.2 |
| ENST00000307921 | 0.00211 | 0.31659 | *ADAT1* | 16 | q23.1 |
| ENST00000379389 | 0.00236 | 0.31326 | *ISG15* | 1 | p36.33 |
| ENST00000239597 | 0.00241 | 0.31270 | *ST3GAL4* | 11 | q24.2 |
| ENST00000299824 | 0.00242 | 0.31256 | *PPP1R16B* | 20 | q11.23 |
| ENST00000382815 | 0.00244 | 0.31233 | *JARID1A* | 12 | p13.33 |
| ENST00000259951 | 0.00249 | 0.31171 | *HLA-F* | 6 | p22.1 |
| ENST00000312426 | 0.00252 | 0.31135 | *NP_076428.2* | 19 | q13.43 |
| ENST00000327435 | 0.00254 | 0.31107 | *ADI1* | 2 | p25.3 |
| ENST00000344836 | 0.00257 | 0.31077 | *USP7* | 16 | p13.2 |
| ENST00000053243 | 0.00264 | 0.30993 | *TNFRSF17* | 16 | p13.13 |
| ENST00000373617 | 0.00266 | 0.30968 | *KPNA6* | 1 | p35.1 |
| ENST00000376818 | 0.00271 | 0.30920 | *HLA-G* | 6 | p22.1 |
| ENST00000292596 | 0.00273 | 0.30894 | *LTC4S* | 5 | q35.3 |
| ENST00000323013 | 0.00274 | 0.30883 | *ZG14* | 22 | q13.2 |
| ENST00000382194 | 0.00279 | 0.30832 | *SMARCA2* | 9 | p24.3 |
| ENST00000377967 | 0.00294 | 0.30671 | *UTX* |  | p11.3 |
| ENST00000383504 | 0.00306 | 0.30555 | *HLAG* | 6 |  |
| ENST00000292510 | 0.00309 | 0.30521 | *VPS28* | 8 | q24.3 |
| ENST00000298532 | 0.00314 | 0.30479 | *SNAPC4* | 9 | q34.3 |
| ENST00000298649 | 0.00325 | 0.30372 | *HK1* | 10 | q22.1 |
| ENST00000255476 | 0.00326 | 0.30364 | *RFXAP* | 13 | q13.3 |
| ENST00000370438 | 0.00326 | 0.30362 | *IDS* | X | q28 |
| ENST00000274787 | 0.00328 | 0.30339 | *HIGD2A* | 5 | q35.2 |
| ENST00000367243 | 0.00329 | 0.30338 | *VIP* | 6 | q25.2 |
| ENST00000369833 | 0.00333 | 0.30298 | *GSTM2* | 1 | p13.3 |
| ENST00000007660 | 0.00343 | 0.30205 | *DLX6* | 7 | q21.3 |
| ENST00000244504 | 0.00354 | 0.30111 | *BTN2A3* | 6 | p22.1 |
| ENST00000278612 | 0.00355 | 0.30097 | *NPAT* | 11 | q22.3 |
| ENST00000369553 | 0.00369 | 0.29985 | *HIPK1* | 1 | p13.2 |
| ENST00000361151 | 0.00378 | 0.29903 | *ZNF266* | 19 | p13.2 |
| ENST00000376043 | 0.00383 | 0.29863 | *TPP2* | 13 | q33.1 |
| ENST00000331835 | 0.00396 | 0.29761 | *SEP15* | 1 | p22.3 |
| ENST00000263688 | 0.00409 | 0.29660 | *C1orf9* | 1 | q24.3 |
| ENST00000195419 | 0.00412 | 0.29638 | *XPO1* | 2 | p15 |
| ENST00000331268 | 0.00413 | 0.29632 | *MORF4L1* | 15 | q25.1 |
| ENST00000355338 | 0.00415 | 0.29619 | *WARS* | 14 | q32.2 |
| ENST00000223298 | 0.00417 | 0.29601 | *TMEM176B* | 7 | q36.1 |
| ENST00000375041 | 0.00423 | 0.29559 | *TRO* | X | p11.21 |
| ENST00000382795 | 0.00430 | 0.29502 | *ARHGEF10* | 8 | p23.3 |
| ENST00000216407 | 0.00433 | 0.29485 | *PSMC6* | 14 | q22.1 |
| ENST00000345541 | 0.00439 | 0.29444 | *WAC* | 10 | p12.1 |
| ENST00000248933 | 0.00444 | 0.29404 | *SEZ6L* | 22 | q12.1 |
| ENST00000359282 | 0.00466 | 0.29251 | *PCBP2* | 12 | q13.13 |
| ENST00000252206 | 0.00468 | 0.29242 | *ZNF165* | 6 | p22.1 |
| ENST00000375440 | 0.00469 | 0.29234 | *CUL4A* | 13 | q34 |
| ENST00000281950 | 0.00481 | 0.29149 | *GEMIN6* | 2 | p22.1 |
| ENST00000342078 | 0.00483 | 0.29142 | *TMEM80* | 11 | p15.5 |
| ENST00000292123 | 0.00485 | 0.29125 | *SAFB* | 19 | p13.3 |
| ENST00000282382 | 0.00491 | 0.29088 | *TICAM2* | 5 | q22.3 |
| ENST00000242770 | 0.00496 | 0.29055 | *STX10* | 19 | p13.13 |
| ENST00000260753 | 0.00515 | 0.28935 | *MPHOSPH1* | 10 | q23.31 |
| ENST00000215861 | 0.00523 | 0.28886 | *CRYBB2* | 22 | q11.23 |
| ENST00000361093 | 0.00525 | 0.28874 | *GART* | 21 | q22.11 |
| ENST00000221114 | 0.00528 | 0.28855 | *DCTN6* | 8 | p12 |
| ENST00000333679 | 0.00528 | 0.28853 | *GAS2L1* | 22 | q12.2 |
| ENST00000349721 | 0.00533 | 0.28823 | *SMARCA2* | 9 | p24.3 |
| ENST00000345739 | 0.00540 | 0.28784 | *ATF2* | 2 | q31.1 |
| ENST00000377764 | 0.00542 | 0.28770 | *HIST1H2BF* | 6 | p22.2 |
| ENST00000311014 | 0.00548 | 0.28736 | *DNAI2* | 17 | q25.1 |
| ENST00000336868 | 0.00551 | 0.28721 | *NXN* | 17 | p13.3 |
| ENST00000244360 | 0.00552 | 0.28713 | *RNF39* | 6 | p21.33 |
| ENST00000367942 | 0.00554 | 0.28701 | *ATF6* | 1 | q23.3 |
| ENST00000377348 | 0.00562 | 0.28653 | *VPS28* | 8 | q24.3 |
| ENST00000221498 | 0.00566 | 0.28633 | *DKKL1* | 19 | q13.33 |
| ENST00000376844 | 0.00569 | 0.28616 | *HLA-F* | 6 | p22.1 |
| ENST00000328649 | 0.00569 | 0.28612 | *CIB1* | 15 | q26.1 |
| ENST00000347635 | 0.00570 | 0.28608 | *NUP50* | 22 | q13.31 |
| ENST00000261253 | 0.00572 | 0.28599 | *JARID1A* | 12 | p13.33 |
| ENST00000340181 | 0.00589 | 0.28501 | *ETV7* | 6 | p21.31 |
| ENST00000276440 | 0.00590 | 0.28499 | *PPP2R2A* | 8 | p21.2 |
| ENST00000298289 | 0.00597 | 0.28457 | *RPL36AL* | 14 | q21.3 |
| ENST00000296581 | 0.00600 | 0.28440 | *LSM6* | 4 | q31.22 |
| ENST00000225724 | 0.00609 | 0.28393 | *GOSR1* | 17 | q11.2 |
| ENST00000250971 | 0.00613 | 0.28371 | *INS* | 11 | p15.5 |
| ENST00000242809 | 0.00615 | 0.28362 | *KLHL24* | 3 | q27.1 |
| ENST00000376663 | 0.00618 | 0.28346 | *BMI1* | 10 | p12.2 |
| ENST00000282903 | 0.00634 | 0.28260 | *PLOD2* | 3 | q24 |
| ENST00000306869 | 0.00635 | 0.28257 | *DCXR* | 17 | q25.3 |
| ENST00000314235 | 0.00640 | 0.28232 | *KCNMB3* | 3 | q26.32 |
| ENST00000374227 | 0.00640 | 0.28232 | *ZFP37* | 9 | q32 |
| ENST00000317615 | 0.00643 | 0.28214 | *TNFSF5IP1* | 18 | p11.21 |
| ENST00000366535 | 0.00644 | 0.28212 | *ADSS* | 1 | q44 |
| ENST00000357471 | 0.00669 | 0.28086 | *PSAP* | 10 | q22.1 |
| ENST00000312293 | 0.00695 | 0.27961 | *FOLR1* | 11 | q13.4 |
| ENST00000370862 | 0.00699 | 0.27940 | *HCRTR2* | 6 | p12.1 |
| ENST00000289041 | 0.00700 | 0.27936 | *XDH* | 2 | p23.1 |
| ENST00000301202 | 0.00705 | 0.27912 | *LAIR2* | 19 | q13.42 |
| ENST00000376861 | 0.00707 | 0.27904 | *HLA-F* | 6 | p22.1 |
| ENST00000336075 | 0.00709 | 0.27893 | *GSTM2* | 1 | p13.3 |
| ENST00000238994 | 0.00709 | 0.27892 | *PPP1R3C* | 10 | q23.32 |
| ENST00000312280 | 0.00710 | 0.27887 | *PMP22* | 17 | p12 |
| ENST00000291568 | 0.00716 | 0.27861 | *CSTB* | 21 | q22.3 |
| ENST00000310473 | 0.00717 | 0.27855 | *UCP2* | 11 | q13.4 |
| ENST00000238613 | 0.00727 | 0.27812 | *C14orf45* | 14 | q24.3 |
| ENST00000258874 | 0.00727 | 0.27812 | *MTHFS* | 15 | q25.1 |
| ENST00000369780 | 0.00727 | 0.27810 | *NEURL* | 10 | q24.33 |
| ENST00000303961 | 0.00728 | 0.27805 | *EGLN2* | 19 | q13.2 |
| ENST00000277465 | 0.00736 | 0.27770 | *CIZ1* | 9 | q34.11 |
| ENST00000370546 | 0.00738 | 0.27762 | *HPSE2* | 10 | q24.2 |
| ENST00000222573 | 0.00742 | 0.27744 | *ITGB8* | 7 | p21.1 |
| ENST00000368922 | 0.00756 | 0.27681 | *DDO* | 6 | q21 |
| ENST00000329683 | 0.00757 | 0.27676 | *NP_000238.1* | 6 | p21.3 |
| ENST00000360686 | 0.00764 | 0.27644 | *Q6ZVX1* | 4 | p16.3 |
| ENST00000356075 | 0.00765 | 0.27642 | *TLK1* | 2 | q31.1 |
| ENST00000235307 | 0.00774 | 0.27604 | *C1orf21* | 1 | q25.3 |
| ENST00000262648 | 0.00774 | 0.27600 | *KAL1* | X | p22.31 |
| ENST00000296255 | 0.00775 | 0.27597 | *RPN1* | 3 | q21.3 |
| ENST00000334399 | 0.00777 | 0.27588 | *HB25* | 6 | p21.32 |
| ENST00000234313 | 0.00780 | 0.27576 | *PLEK* | 2 | p13.3 |
| ENST00000337318 | 0.00781 | 0.27573 | *FAM53B* | 10 | q26.13 |
| ENST00000287968 | 0.00786 | 0.27548 | *NP_001034792.1* | 1 | p36.13 |
| ENST00000262450 | 0.00793 | 0.27522 | *CHD5* | 1 | p36.31 |
| ENST00000252457 | 0.00800 | 0.27493 | *CDC16* | 13 | q34 |
