# Supplementary material for: Concordant Gene Expression in Leukemia Cells and Normal Leukocytes Is Associated with Germline cis-SNPs
Source: PLoS One. 2008 May 14;3(5):e2144. doi: 10.1371/journal.pone.0002144 (PMC2374895; doi:10.1371/journal.pone.0002144)
Supplement: Table S2 — 31 “housekeeping genes” whose expression is concordant between diagnostic leukemia cells and normal peripheral blood leukocytes (0.07 MB DOC) [file pone.0002144.s002.doc]

Table S2: 31 “housekeeping genes” whose expression is concordant between diagnostic leukemia cells and normal peripheral blood leukocytes[1].

| **Gene symbol** | **Chromosome** | **Description** |
| --- | --- | --- |
| *C2orf17* | 2q35 | Chromosome 2 open reading frame 17 |
| *CIB1* | Top of Form  15q25.3-q26 | Top of Form  calcium and integrin binding 1 (calmyrin) |
| *CSTB* | Top of Form  21q22.3 | Top of Form  cystatin B (stefin B) |
| *CTBP1* | Top of Form  4p16 | Top of Form  C-terminal binding protein 1 |
| *DCTN6* | Top of Form  8p12-p11 | Top of Form  dynactin 6 |
| *DDX17* | Top of Form  22q13.1 | DEAD (Asp-Glu-Ala-Asp) box polypeptide 17 |
| *EIF5AP1* | Top of Form  10q23.3 | Top of Form  eukaryotic translation initiation factor 5A pseudogene 1 |
| *FAM53B* | Top of Form  10q26.13 | Top of Form  family with sequence similarity 53, member B |
| *GAPDH* | Top of Form  12p13 | glyceraldehyde-3-phosphate dehydrogenase |
| *GLG1* | Top of Form  16q22-q23 | Top of Form  golgi apparatus protein 1 |
| *GRHPR* | Top of Form  9q12 | Top of Form  glyoxylate reductase/hydroxypyruvate reductase |
| *GSTM2* | Top of Form  1p13.3 | Top of Form  glutathione S-transferase M2 |
| *HIGD2A* | Top of Form  5q35.2 | Top of Form  HIG1 domain family, member 2A |
| *HLA-G* | Top of Form  6p21.3 | Top of Form  HLA-G histocompatibility antigen, class I, G |
| *KPNA6* | Top of Form  1p35.1-p34.3 | karyopherin alpha 6 (importin alpha 7) |
| *MORF4L1* | Top of Form  15q24 | Top of Form  mortality factor 4 like 1 |
| *NCOR1* | Top of Form  17p11.2 | Top of Form  nuclear receptor co-repressor 1 |
| *PCBP2* | Top of Form  12q13.12-q13.13 | Top of Form  poly(rC) binding protein 2 |
| *PPP2R2A* | Top of Form  8p21.2 | Top of Form  protein phosphatase 2, regulatory subunit B, alpha isoform |
| *PSAP* | Top of Form  10q21-q22 | Top of Form  prosaposin |
| *PSMA6* | Top of Form  14q13 | Top of Form  proteasome (prosome, macropain) subunit, alpha type, 6 |
| *PSME3* | Top of Form  17q21 | Top of Form  proteasome (prosome, macropain) activator subunit 3 |
| *RPL36AL* | Top of Form  14q21 | Top of Form  ribosomal protein L36a-like |
| *RPS12* | Top of Form  6q23.2 | ribosomal protein S12 |
| *TNFSF5IP1* | Top of Form  18p11.21 | Top of Form  tumor necrosis factor superfamily, member 5-induced protein 1 |
| *UCP2* | Top of Form  11q13 | Top of Form  uncoupling protein 2 (mitochondrial, proton carrier) |
| *WAC* | 10p11.23 | Top of Form  WW domain containing adaptor with coiled-coil |
| *WARS* | Top of Form  14q32.31 | Top of Form  tryptophanyl-tRNA synthetase |
| *XDH* | Top of Form  2p23.1 | Top of Form  xanthine dehydrogenase |
| *XPO1* | Top of Form  2p16 | Top of Form  exportin 1 |
| *ZRSR1* | Top of Form  5q22 | Top of Form  zinc finger (CCCH type), RNA-binding motif and serine/arginine rich 1 |

Reference List

1 Tu Z, Wang L, Xu M, Zhou X, Chen T et al. (2006) Further understanding human disease genes by comparing with housekeeping genes and other genes. BMC Genomics 7: 31.
