# Supplementary material for: Concordant Gene Expression in Leukemia Cells and Normal Leukocytes Is Associated with Germline cis-SNPs
Source: PLoS One. 2008 May 14;3(5):e2144. doi: 10.1371/journal.pone.0002144 (PMC2374895; doi:10.1371/journal.pone.0002144)
Supplement: Table S3 — Using MEME, 3 DNA sequence motifs were found to be overrepresented in the 20 genes whose expression was concordant between leukemia cells and normal leukocytes and that had cis-SNPs associated with their expression. These motifs were also found in the genes in the leukemia cells (green shading) and normal leukocytes (yellow shading) that had cis-SNPs associated with their expression, but whose expression was not concordant in these two tissues. None of the motifs are known transcription factor binding sites. (0.03 MB DOC) [file pone.0002144.s003.doc]

Table S3: Using MEME, 3 DNA sequence motifs were found to be overrepresented in the 20 genes whose expression was concordant between leukemia cells and normal leukocytes and that had *cis*-SNPs associated with their expression. These motifs were also found in the genes in the leukemia cells (green shading) and normal leukocytes (yellow shading) that had *cis*-SNPs associated with their expression, but whose expression was not concordant in these two tissues. None of the motifs are known transcription factor binding sites.

|  | # of *cis*-SNPs | Motif 1  TCTCTGTGTTCTTTT | Motif 2  TCCCTCTGCCCCTGG | Motif 3  TTGCTGGG |
| --- | --- | --- | --- | --- |
| 20 *cis*-SNP genes | 86 | 62 | 49 | 23 |
| 532 leukemia cell genes with *cis*-SNPs * | 939 | 86 | 80 | 14 |
| 559 normal leukocyte genes with *cis*-SNPs ^#^ | 1072 | 86 | 86 | 14 |

* Motifs 1, 2 and 3 are found at a significantly lower frequency in the leukemia cell genes (green shading) with *cis*-SNPs predicting their expression than in the 20 *cis*-SNP genes whose expression was concordant between tissues (p=2.28x10^-43^, 1.64x10^-25^  and 2.96x10^-17^ respectively, Fishers exact test).

^#^ Motifs 1, 2 and 3 are found at a significantly lower frequency in the normal leukocyte genes (yellow shading) with *cis*-SNPs predicting their expression than in the 20 *cis*-SNP genes whose expression was concordant between tissues (p=1.07x10^-46^, 7.06x10^-27^  and 1.94x10^-18^ respectively, Fishers exact test).
