# Supplementary material for: Concordant Gene Expression in Leukemia Cells and Normal Leukocytes Is Associated with Germline cis-SNPs
Source: PLoS One. 2008 May 14;3(5):e2144. doi: 10.1371/journal.pone.0002144 (PMC2374895; doi:10.1371/journal.pone.0002144)
Supplement: Figure S1 — Unsupervised hierarchical clustering of gene expression for 184 samples (columns) from 92 patients indicates two distinct clusters which segregate the different tissue types: diagnostic leukemia cells and normal leukocytes. (Blood: normal leukocyte samples; BH: B-lineage ALL with hyperdiploid karyotype; BN: B-lineage ALL with non-hyperdiploid karyotype; bcr: BCR-ABL translocation; e2a: E2A-PBX translocation; mll: MLL-AF4 fusion; other: B-lineage ALL with no defined translocations; tel: TEL-AML1 translocation; T: T-lineage ALL). (0.05 MB DOC) [file pone.0002144.s004.doc]

Figure S1: Unsupervised hierarchical clustering of gene expression for 184 samples (columns) from 92 patients indicates two distinct clusters which segregate the different tissue types: diagnostic leukemia cells and normal leukocytes. (Blood: normal leukocyte samples; BH: B-lineage ALL with hyperdiploid karyotype; BN: B-lineage ALL with non-hyperdiploid karyotype; bcr: *BCR-ABL* translocation; e2a: *E2A-PBX* translocation; mll: *MLL-AF4* fusion; other: B-lineage ALL with no defined translocations; tel: *TEL-AML1* translocation; T: T-lineage ALL).
