# Supplementary material for: Concordant Gene Expression in Leukemia Cells and Normal Leukocytes Is Associated with Germline cis-SNPs
Source: PLoS One. 2008 May 14;3(5):e2144. doi: 10.1371/journal.pone.0002144 (PMC2374895; doi:10.1371/journal.pone.0002144)
Supplement: Figure S2 — Each circle represents a probe set. The average expression of each probe set in diagnostic leukemia cells for the 92 patients is shown on the x-axis and for normal leukocytes is shown on the y-axis. The line of identity shows comparable expression levels in both cell types. The blue circles indicate the 204 probe sets whose expression was concordant between the diagnostic leukemia cells and the normal leukocytes. The red circles indicate the 21 of these 204 probe sets that had cis-SNPs predicting their expression. The grey circles are the remaining 14,915 probe sets. The closer the circles are to the line of identity, the more similar the level of expression of that probe set in both tissues. The dotted line indicates a log2 expression level of 8, indicating that the 21 probe sets with cis-SNPs predicting their expression is higher than the overall expression of the 204 probe sets whose expression is concordant between tissue types. (2.06 MB DOC) [file pone.0002144.s005.doc]

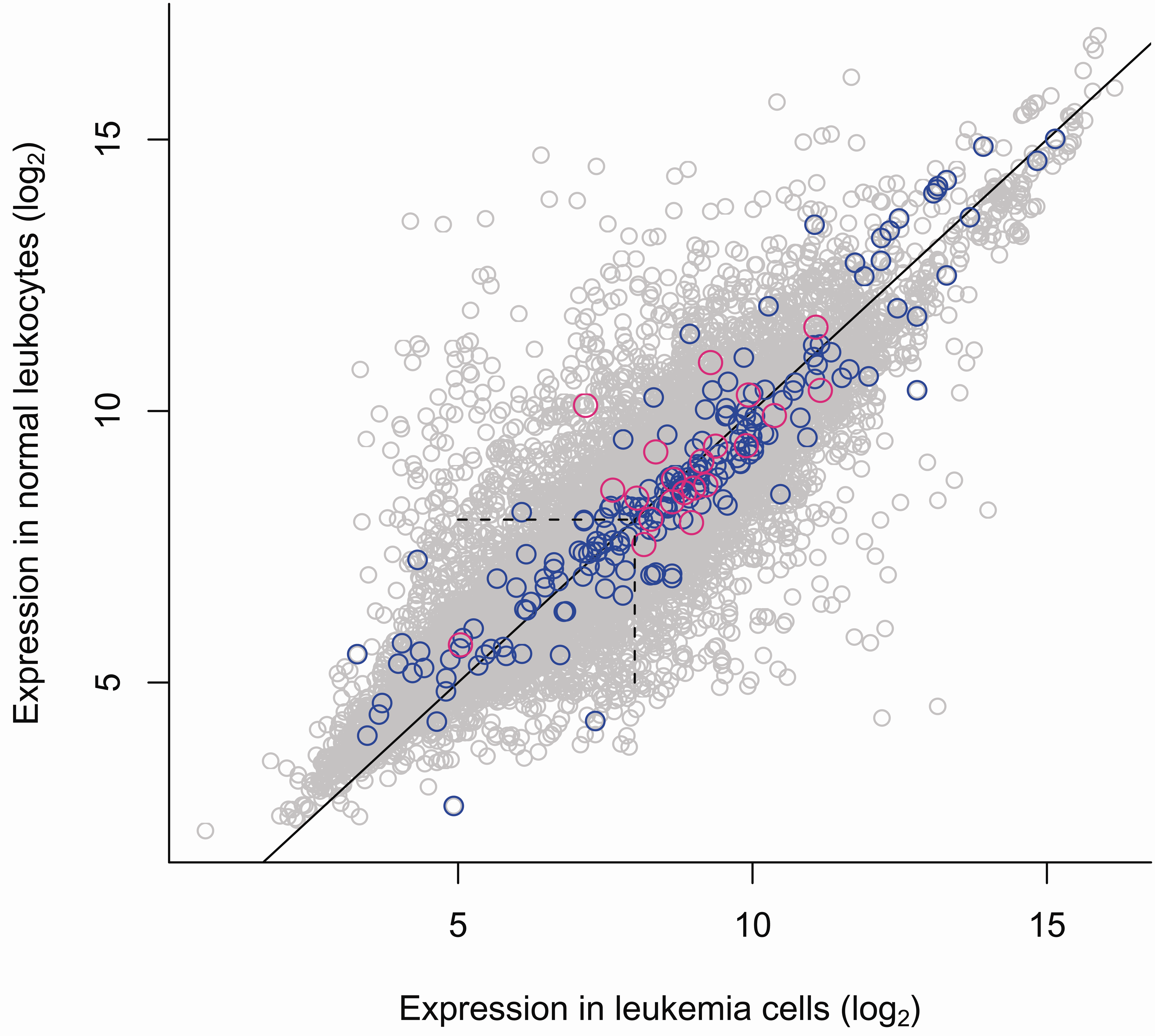
Figure S2: Each circle represents a probe set. The average expression of each probe set in diagnostic leukemia cells for the 92 patients is shown on the x-axis and for normal leukocytes is shown on the y-axis. The line of identity shows comparable expression levels in both cell types. The blue circles indicate the 204 probe sets whose expression was concordant between the diagnostic leukemia cells and the normal leukocytes. The red circles indicate the 21 of these 204 probe sets that had *cis*-SNPs predicting their expression. The grey circles are the remaining 14,915 probe sets. The closer the circles are to the line of identity, the more similar the level of expression of that probe set in both tissues. The dotted line indicates a log_2_ expression level of 8, indicating that the 21 probe sets with *cis*-SNPs predicting their expression is higher than the overall expression of the 204 probe sets whose expression is concordant between tissue types.
