# Supplementary material for: Concordant Gene Expression in Leukemia Cells and Normal Leukocytes Is Associated with Germline cis-SNPs
Source: PLoS One. 2008 May 14;3(5):e2144. doi: 10.1371/journal.pone.0002144 (PMC2374895; doi:10.1371/journal.pone.0002144)
Supplement: Figure S3 — a) Histogram showing the number of probe sets (y-axis) whose expression was correlated between tissue types at specific p-value cut offs (x-axis). The histogram indicates a significant correlation between gene expression in diagnostic leukemia cells and normal leukocytes as shown by an over-representation of probe sets with low p-values. A positive correlation of gene expression between diagnostic leukemia cells and normal leukocytes is shown by white shading and a negative correlation is shown by grey shading. The figure indicates a significantly higher number of probe sets than expected are positively correlated between diagnostic leukemia cells and normal leukocytes. b) Hierarchical clustering of gene expression with each column representing one of the 184 patient samples (92 leukemia cell and 92 normal leukocyte samples) and each row representing one of the top 136 probe sets (ordered by p-value; p<0.0048 cut-off) that are positively correlated between the diagnostic leukemia cells and normal leukocytes. Using these 136 probe sets, 55 out of the 92 patient sample pairs were clustered together as indicated with blue dashes below the clustering. Green = low expression and red = high expression. (0.12 MB DOC) [file pone.0002144.s006.doc]

Figure S3: a) Histogram showing the number of probe sets (y-axis) whose expression was correlated between tissue types at specific p-value cut offs (x-axis). The histogram indicates a significant correlation between gene expression in diagnostic leukemia cells and normal leukocytes as shown by an over-representation of probe sets with low p-values. A positive correlation of gene expression between diagnostic leukemia cells and normal leukocytes is shown by white shading and a negative correlation is shown by grey shading. The figure indicates a significantly higher number of probe sets than expected are positively correlated between diagnostic leukemia cells and normal leukocytes.


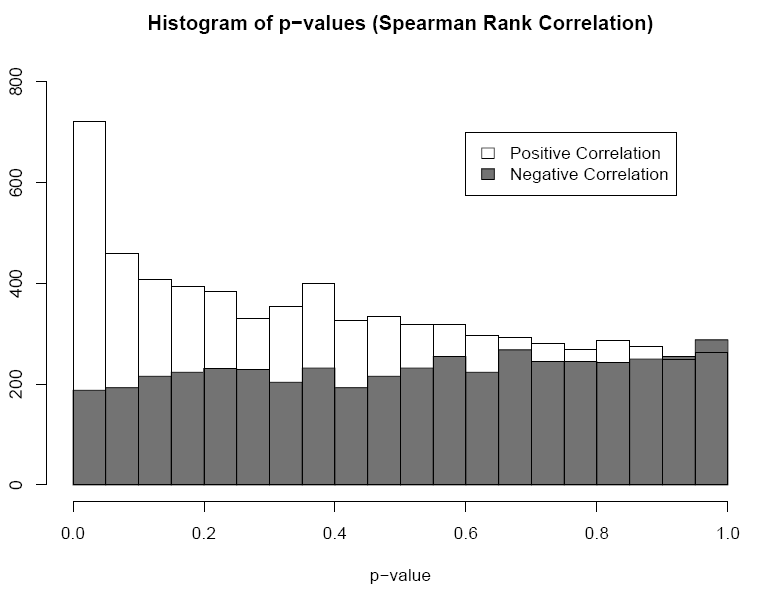


b) Hierarchical clustering of gene expression with each column representing one of the 184 patient samples (92 leukemia cell and 92 normal leukocyte samples) and each row representing one of the top 136 probe sets (ordered by p-value; p<0.0048 cut-off) that are positively correlated between the diagnostic leukemia cells and normal leukocytes. Using these 136 probe sets, 55 out of the 92 patient sample pairs were clustered together as indicated with blue dashes below the clustering. Green = low expression and red= high expression.
