# Supplementary material for: Concordant Gene Expression in Leukemia Cells and Normal Leukocytes Is Associated with Germline cis-SNPs
Source: PLoS One. 2008 May 14;3(5):e2144. doi: 10.1371/journal.pone.0002144 (PMC2374895; doi:10.1371/journal.pone.0002144)
Supplement: Figure S4 — a) Examples of genes (here ADI1, PEX6, AMFR, ZNF266 and SAR1A) whose expression was concordant between diagnostic leukemia cells and normal leukocytes: Left: expression levels among 92 patients in leukemia cells and normal leukocytes are correlated (p = 0.0025, <0.00001, 1.44×10^−8^, 0.0038, <0.00001 respectively); Middle: expression of these genes (median, quantiles, range) in leukemia cells was associated (adjusted p = 0.0016, 0.0006, 0.0002, 0.0004, 0.001 respectively) with the germline genotype of a particular cis-SNP in this gene; Right: expression of these genes in normal leukocytes was associated (adjusted p = 0.0072, 0.0006, 0.0014, 0.0004, 0.0208 respectively) with the germline genotype at the same cis-SNP. b) Examples of genes (here NRG1, NCOA1, C20orf23, C9orf39, ATM) whose expression was not concordant between diagnostic leukemia cells and normal leukocytes: Left: expression levels among 92 patients in leukemia cells and normal leukocytes was not concordant (p = 0.9799, 0.9377, 0.9523, 0.9128, 0.9607 respectively); Middle: expression of these genes in leukemia cells was not associated (adjusted p = 0.9996, 0.080, 1.000, 0.5106, 0.9996 respectively) with the germline genotype of a particular cis-SNP in this gene; Right: expression of these genes in normal leukocytes was not associated (adjusted p = 1.000, 1.000, 1.000, 0.9982, 0.9320 respectively) with the germline genotype at the same cis-SNP. (0.20 MB DOC) [file pone.0002144.s007.doc]

Figure S4: a) Examples of genes (here *ADI1*, *PEX6*, *AMFR*, *ZNF266* and *SAR1A*) whose expression was concordant between diagnostic leukemia cells and normal leukocytes: Left: expression levels among 92 patients in leukemia cells and normal leukocytes are correlated (p=0.0025, <0.00001, 1.44x10^-8^, 0.0038, <0.00001 respectively); Middle: expression of these genes (median, quantiles, range) in leukemia cells was associated (adjusted p=0.0016, 0.0006, 0.0002, 0.0004, 0.001 respectively) with the germline genotype of a particular *cis*-SNP in this gene; Right: expression of these genes in normal leukocytes was associated (adjusted p=0.0072, 0.0006, 0.0014, 0.0004, 0.0208 respectively) with the germline genotype at the same *cis*-SNP.

b) Examples of genes (here *NRG1, NCOA1, C20orf23, C9orf39, ATM*) whose expression was not concordant between diagnostic leukemia cells and normal leukocytes: Left: expression levels among 92 patients in leukemia cells and normal leukocytes was not concordant (p=0.9799, 0.9377, 0.9523, 0.9128, 0.9607 respectively); Middle: expression of these genes in leukemia cells was not associated (adjusted p=0.9996, 0.080, 1.000, 0.5106, 0.9996 respectively) with the germline genotype of a particular *cis*-SNP in this gene; Right: expression of these genes in normal leukocytes was not associated (adjusted p=1.000, 1.000, 1.000, 0.9982, 0.9320 respectively) with the germline genotype at the same *cis*-SNP.
