# Supplementary material for: Concordant Gene Expression in Leukemia Cells and Normal Leukocytes Is Associated with Germline cis-SNPs
Source: PLoS One. 2008 May 14;3(5):e2144. doi: 10.1371/journal.pone.0002144 (PMC2374895; doi:10.1371/journal.pone.0002144)

Figure S5: a) At adjusted p<0.05, there was no difference in the number of *cis*-SNPs associated with the expression of the 176 concordant genes in the normal leukocytes and the leukemia cells that constituted our discovery set. 133 of these 176 concordantly expressed genes are represented on the Exon Array for the HapMap cell lines that constituted one validation set. There was no significant difference between the number of *cis*-SNPs associated with the expression of these 133 genes in the HapMap cell lines (validation set) compared to the number of *cis*-SNPs associated with expression of the 176 genes in the normal leukocytes (p=0.34, Fishers exact test) or the diagnostic leukemia cells (p=0.34, Fishers exact test). Specifically, in the normal leukocytes (yellow circle), there are 30 genes with significant *cis*-SNPs; in the leukemia cells (green circle), there are 30 genes with significant *cis*-SNPs; and in the HapMap cell lines (pink circle), there are 17 genes with significant *cis*-SNPs, focusing on the genes whose expression was concordant between leukemia cells and normal leukocytes. There are 8 out of 176 genes (centre of VENN diagram) with significant *cis*-SNPs that are common to all 3 sets representing 4.5%^*^ of the 176 genes.


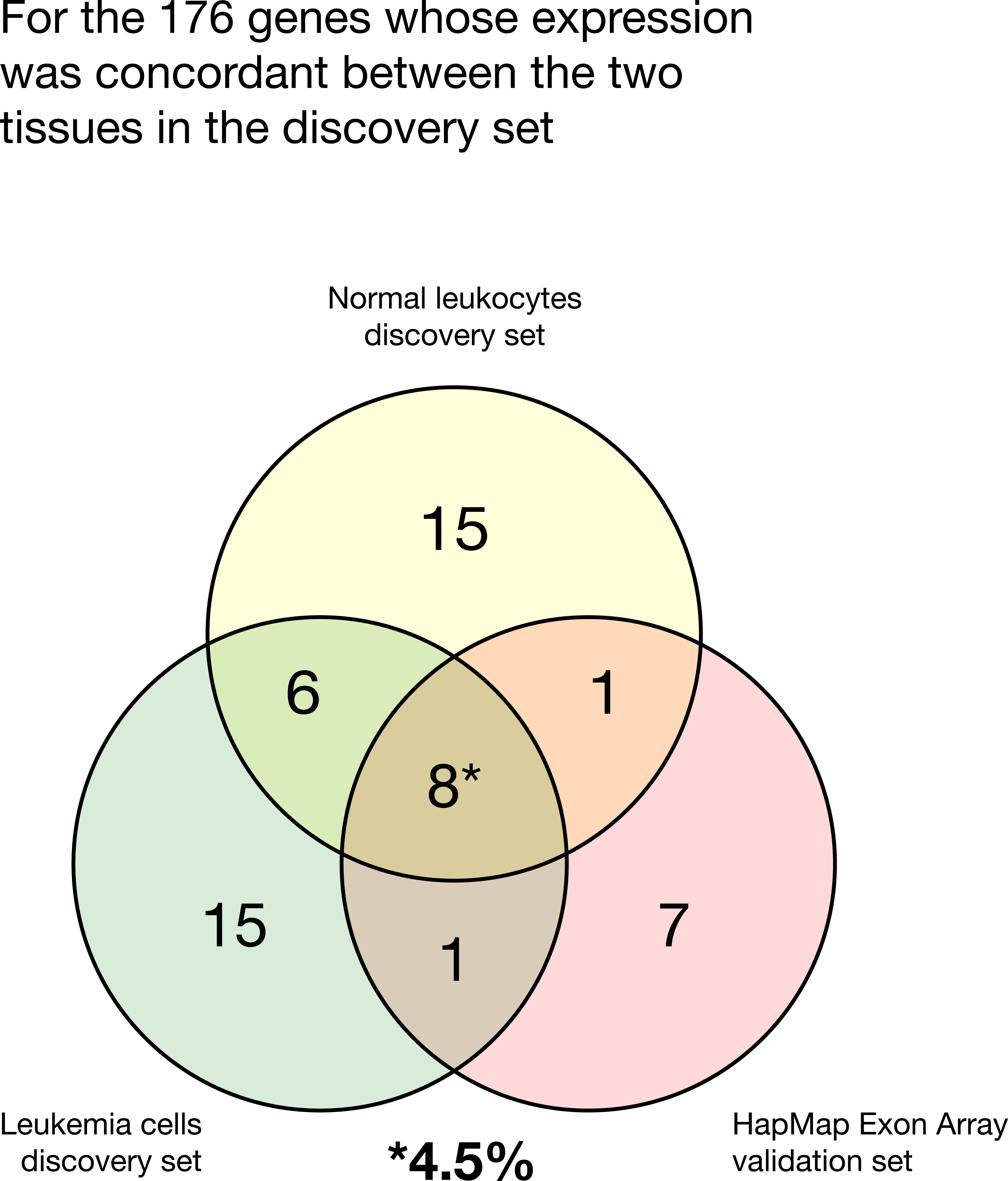


b) At adjusted p<0.05, there was no difference in the number of *cis*-SNPs associated with the expression of the 8,853 expressed genes in the normal leukocytes and the leukemia cells that constituted one discovery set. 8,187 of the 8,853 expressed genes are represented on the Exon Array for the HapMap cell lines that constituted our validation set. There was a significant difference between the number of *cis*-SNPs associated with the expression of these 8,187 genes in the HapMap cell lines (validation set) compared to the number of *cis*-SNPs associated with expression of the 8,853 genes in the normal leukocytes (p=1.30x10^-11^, Fishers exact test) and the diagnostic leukemia cells (p=3.00x10^-14^, Fishers exact test). Specifically, in the normal leukocytes (yellow circle), there are 559 genes with significant *cis*-SNPs; in the leukemia cells (green circle), there are 532 genes with significant *cis*-SNPs; and in the HapMap cell lines (pink circle), there are 743 genes with significant *cis*-SNPs, whose expression was not concordant between tissues. There are 15 out of 8,853 genes (center of VENN diagram) with significant *cis*-SNPs common to all 3 sets representing 0.17%^*^ of the 8,853 genes.


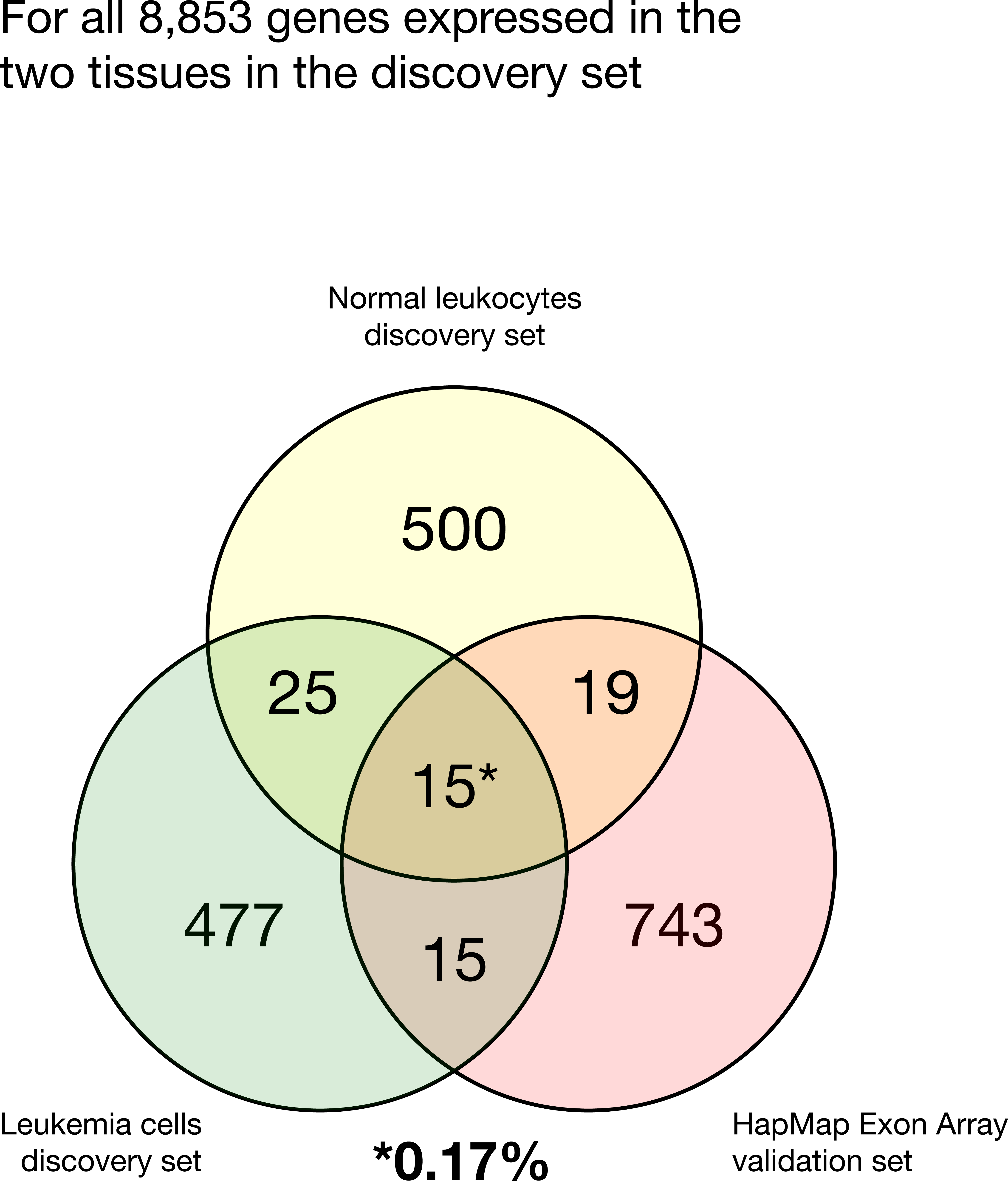

Supplement: Figure S5 — a) At adjusted p<0.05, there was no difference in the number of cis-SNPs associated with the expression of the 176 concordant genes in the normal leukocytes and the leukemia cells that constituted our discovery set. 133 of these 176 concordantly expressed genes are represented on the Exon Array for the HapMap cell lines that constituted one validation set. There was no significant difference between the number of cis-SNPs associated with the expression of these 133 genes in the HapMap cell lines (validation set) compared to the number of cis-SNPs associated with expression of the 176 genes in the normal leukocytes (p = 0.34, Fishers exact test) or the diagnostic leukemia cells (p = 0.34, Fishers exact test). Specifically, in the normal leukocytes (yellow circle), there are 30 genes with significant cis-SNPs; in the leukemia cells (green circle), there are 30 genes with significant cis-SNPs; and in the HapMap cell lines (pink circle), there are 17 genes with significant cis-SNPs, focusing on the genes whose expression was concordant between leukemia cells and normal leukocytes. There are 8 out of 176 genes (centre of VENN diagram) with significant cis-SNPs that are common to all 3 sets representing 4.5%* of the 176 genes. b) At adjusted p<0.05, there was no difference in the number of cis-SNPs associated with the expression of the 8,853 expressed genes in the normal leukocytes and the leukemia cells that constituted one discovery set. 8,187 of the 8,853 expressed genes are represented on the Exon Array for the HapMap cell lines that constituted our validation set. There was a significant difference between the number of cis-SNPs associated with the expression of these 8,187 genes in the HapMap cell lines (validation set) compared to the number of cis-SNPs associated with expression of the 8,853 genes in the normal leukocytes (p = 1.30×10^−11^, Fishers exact test) and the diagnostic leukemia cells (p = 3.00×10^−14^, Fishers exact test). Specifically, in the normal leukocy [file pone.0002144.s008.doc]
