# Supplementary material for: Concordant Gene Expression in Leukemia Cells and Normal Leukocytes Is Associated with Germline cis-SNPs
Source: PLoS One. 2008 May 14;3(5):e2144. doi: 10.1371/journal.pone.0002144 (PMC2374895; doi:10.1371/journal.pone.0002144)

Figure S6: Results of pathway analysis of genes whose expression was concordant between leukemia cells and normal leukocytes and had *cis*-SNPs associated with their expression in the discovery set (n=20) compared to those genes that are expressed, but not concordantly expressed in the two tissues and have *cis*-SNPs associated with their expression (n=1574), as determined by interrogating the Database for Annotation, Visualization and Integrated Discovery (DAVID; <http://david.abcc.ncifcrf.gov/>). None of the pathways reached statistical significance using this tool.


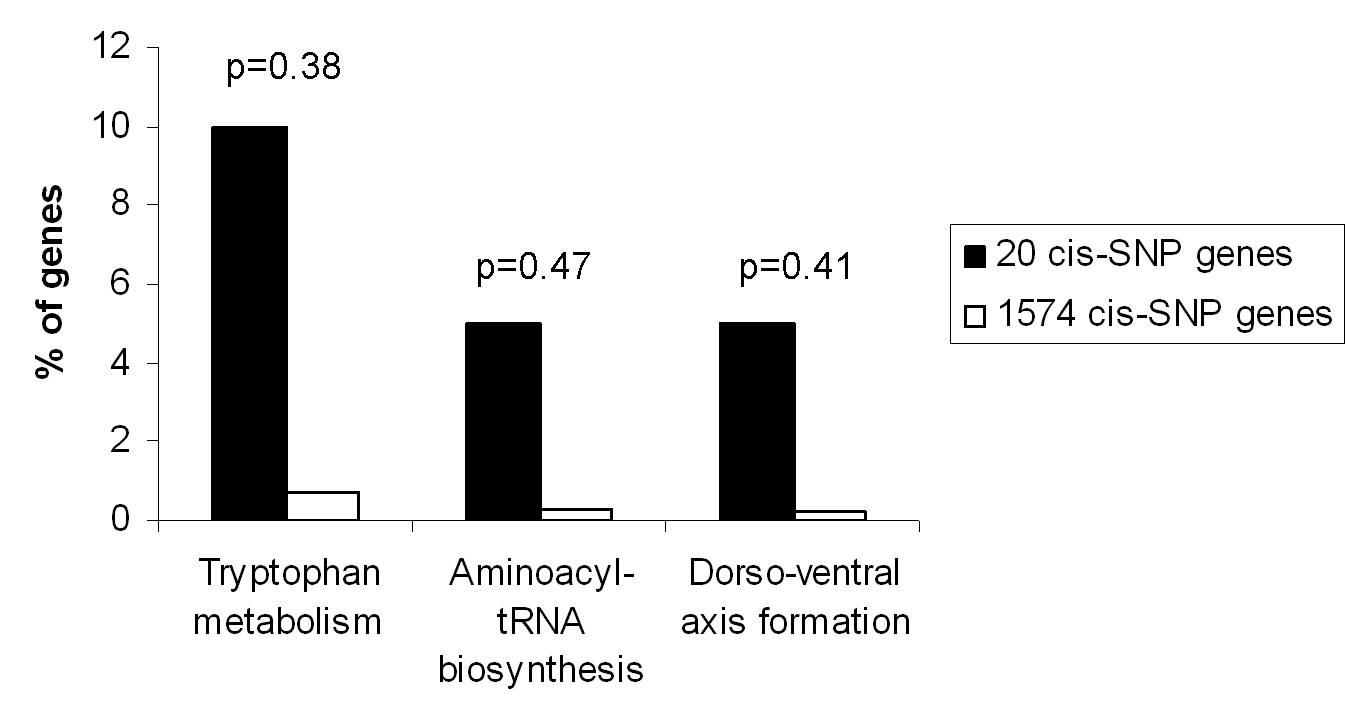

Supplement: Figure S6 — Results of pathway analysis of genes whose expression was concordant between leukemia cells and normal leukocytes and had cis-SNPs associated with their expression in the discovery set (n = 20) compared to those genes that are expressed, but not concordantly expressed in the two tissues and have cis-SNPs associated with their expression (n = 1574), as determined by interrogating the Database for Annotation, Visualization and Integrated Discovery (DAVID; http://david.abcc.ncifcrf.gov/). None of the pathways reached statistical significance using this tool. (0.08 MB DOC) [file pone.0002144.s009.doc]
