# Supplementary material for: Concordant Gene Expression in Leukemia Cells and Normal Leukocytes Is Associated with Germline cis-SNPs
Source: PLoS One. 2008 May 14;3(5):e2144. doi: 10.1371/journal.pone.0002144 (PMC2374895; doi:10.1371/journal.pone.0002144)

Figure S7: Results of pathway analysis of genes whose expression was concordant between leukemia cells and normal leukocytes in the discovery set and had *cis*-SNPs affecting their expression (n=20) to over represent specific pathways compared to those genes that did not have *cis*-SNPs associated with their expression (n=156) as determined by interrogating DAVID. Genes involved in the tryptophan metabolism pathway were marginally significantly over represented using this tool (p=0.05, Fishers exact test).


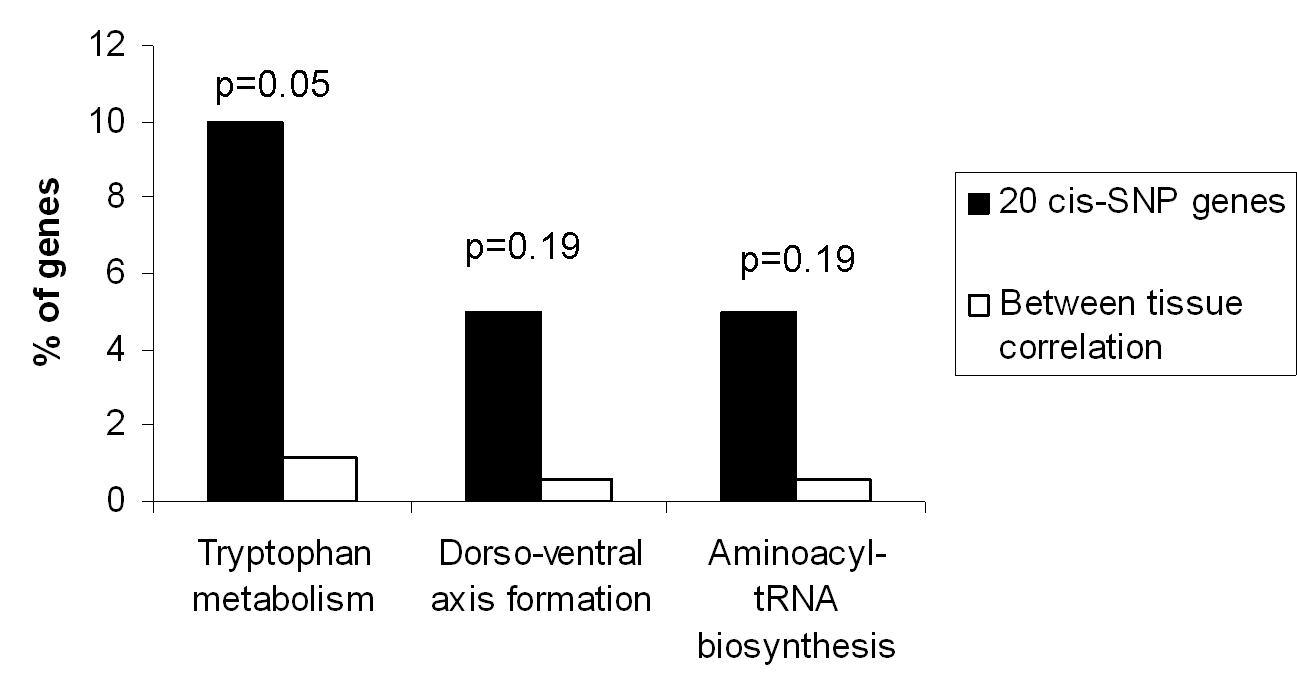

Supplement: Figure S7 — Results of pathway analysis of genes whose expression was concordant between leukemia cells and normal leukocytes in the discovery set and had cis-SNPs affecting their expression (n = 20) to over represent specific pathways compared to those genes that did not have cis-SNPs associated with their expression (n = 156) as determined by interrogating DAVID. Genes involved in the tryptophan metabolism pathway were marginally significantly over represented using this tool (p = 0.05, Fishers exact test). (0.07 MB DOC) [file pone.0002144.s010.doc]
