# Supplementary material for: Concordant Gene Expression in Leukemia Cells and Normal Leukocytes Is Associated with Germline cis-SNPs
Source: PLoS One. 2008 May 14;3(5):e2144. doi: 10.1371/journal.pone.0002144 (PMC2374895; doi:10.1371/journal.pone.0002144)

Figure S8: There was no strong correlation (p>0.05, Pearson’s correlation) in the diagnostic leukemia cells of expression between the 20 *cis*-SNP genes whose expression is concordant between diagnostic leukemia cells and normal leukocytes. Yellow indicates a strong positive correlation and increasing blue indicates no strong positive correlation.


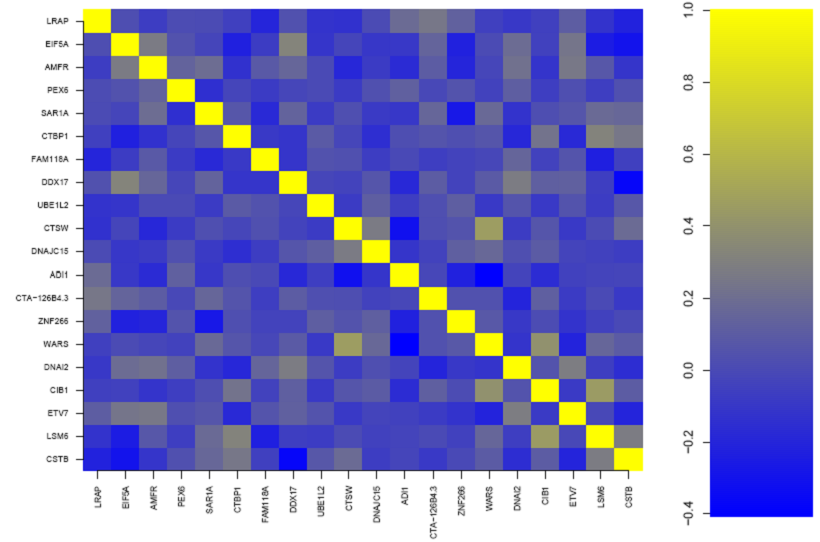

Supplement: Figure S8 — There was no strong correlation (p>0.05, Pearson's correlation) in the diagnostic leukemia cells of expression between the 20 cis-SNP genes whose expression is concordant between diagnostic leukemia cells and normal leukocytes. Yellow indicates a strong positive correlation and increasing blue indicates no strong positive correlation. (0.10 MB DOC) [file pone.0002144.s011.doc]
