# Supplementary material for: Concordant Gene Expression in Leukemia Cells and Normal Leukocytes Is Associated with Germline cis-SNPs
Source: PLoS One. 2008 May 14;3(5):e2144. doi: 10.1371/journal.pone.0002144 (PMC2374895; doi:10.1371/journal.pone.0002144)
Supplement: Figure S10 — There was no significant difference between the number of cis-SNPs per gene in the 20 genes whose expression was concordant between tissue types and that had cis-SNPs associated with their expression and the 8,853 genes whose expression was not concordant (p = 0.769, Wilcoxon test). (0.06 MB DOC) [file pone.0002144.s013.doc]

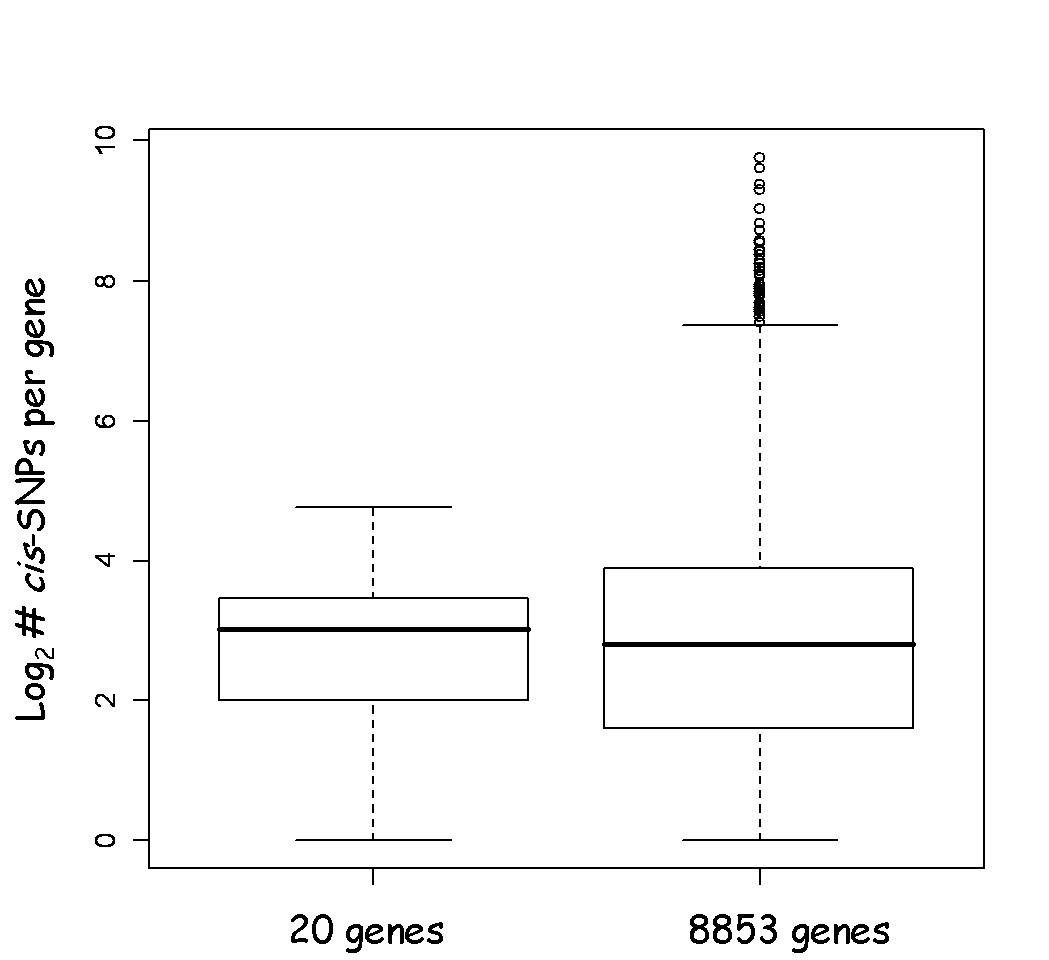
Figure S10: There was no significant difference between the number of *cis*-SNPs per gene in the 20 genes whose expression was concordant between tissue types and that had *cis*-SNPs associated with their expression and the 8,853 genes whose expression was not concordant (p=0.769, Wilcoxon test).
